# Downregulation of tRF-Cys-GCA-029 by hyperglycemia promotes tumorigenesis and glycolysis of diabetic breast cancer through upregulating PRKCG translation

**DOI:** 10.1186/s13058-024-01870-1

**Published:** 2024-07-22

**Authors:** Yongyi Huang, Cheng Chen, Yang Liu, Binbin Tan, Qin Xiang, Qianqian Chen, Yiling Wang, Wenhan Yang, Jingsong He, Duanyang Zhou, Yuting Wang, Kaiping Gao, Duo Zheng, Rihong Zhai

**Affiliations:** 1 School of Public Health, Guangdong Key Laboratory for Genome Stability & Disease Prevention, International Cancer Center, Shenzhen University Medical School, Shenzhen, 518055 China; 2 Department of Cell Biology, Shenzhen University Medical School, Shenzhen, 518055 China; 3 Department of Surgery, Cancer Hospital of Harbin Medical University, Harbin, 150081 China; 4 Department of Breast Surgery, Peking University Shenzhen Hospital, 1120 Lianhua Road, Shenzhen, 518036 China

**Keywords:** Diabetic breast cancer, Hyperglycaemia, tRF-Cys-GCA-029, PRKCG, Glycolysis

## Abstract

**Background:**

Diabetes mellitus (DM) affects up to one-third of breast cancer (BC) patients. Patients with co-existing BC and DM (BC-DM) have worsened BC prognosis. Nevertheless, the molecular mechanisms orchestrating BC-DM prognosis remain poorly understood. tRNA-derived fragments (tRFs) have been shown to regulate cancer progression. However, the biological role of tRFs in BC-DM has not been explored.

**Methods:**

tRF levels in tumor tissues and cells were detected by tRF sequencing and qRT-PCR. The effects of tRF on BC cell malignancy were assessed under euglycemic and hyperglycemic conditions in vitro. Metabolic changes were assessed by lactate, pyruvate, and extracellular acidification rate (ECAR) assays. Diabetic animal model was used to evaluate the impacts of tRF on BC tumor growth. RNA-sequencing (RNA-seq), qRT-PCR, Western blot, polysome profiling, luciferase reporter assay, and rescue experiments were performed to explore the regulatory mechanisms of tRF in BC-DM.

**Results:**

We identified that tRF-Cys-GCA-029 was downregulated in BC-DM tissues and under hyperglycemia conditions in BC cells. Functionally, downregulation of tRF-Cys-GCA-029 promoted BC cell proliferation and migration in a glucose level-dependent manner. tRF-Cys-GCA-029 knockdown also enhanced glycolysis metabolism in BC cells, indicated by increasing lactate/pyruvate production and ECAR levels. Notably, injection of tRF-Cys-GCA-029 mimic significantly suppressed BC tumor growth in diabetic-mice. Mechanistically, tRF-Cys-GCA-029 regulated BC cell malignancy and glycolysis via interacting with PRKCG in two ways: binding to the coding sequence (CDS) of PRKCG mRNA to regulate its transcription and altering polysomal PRKCG mRNA expression to modify its translation.

**Conclusions:**

Hyperglycemia-downregulated tRF-Cys-GCA-029 enhances the malignancy and glycolysis of BC cells. tRF-Cys-GCA-029-PRKCG-glycolysis axis may be a potential therapeutic target against BC-DM.

**Graphical Abstract:**

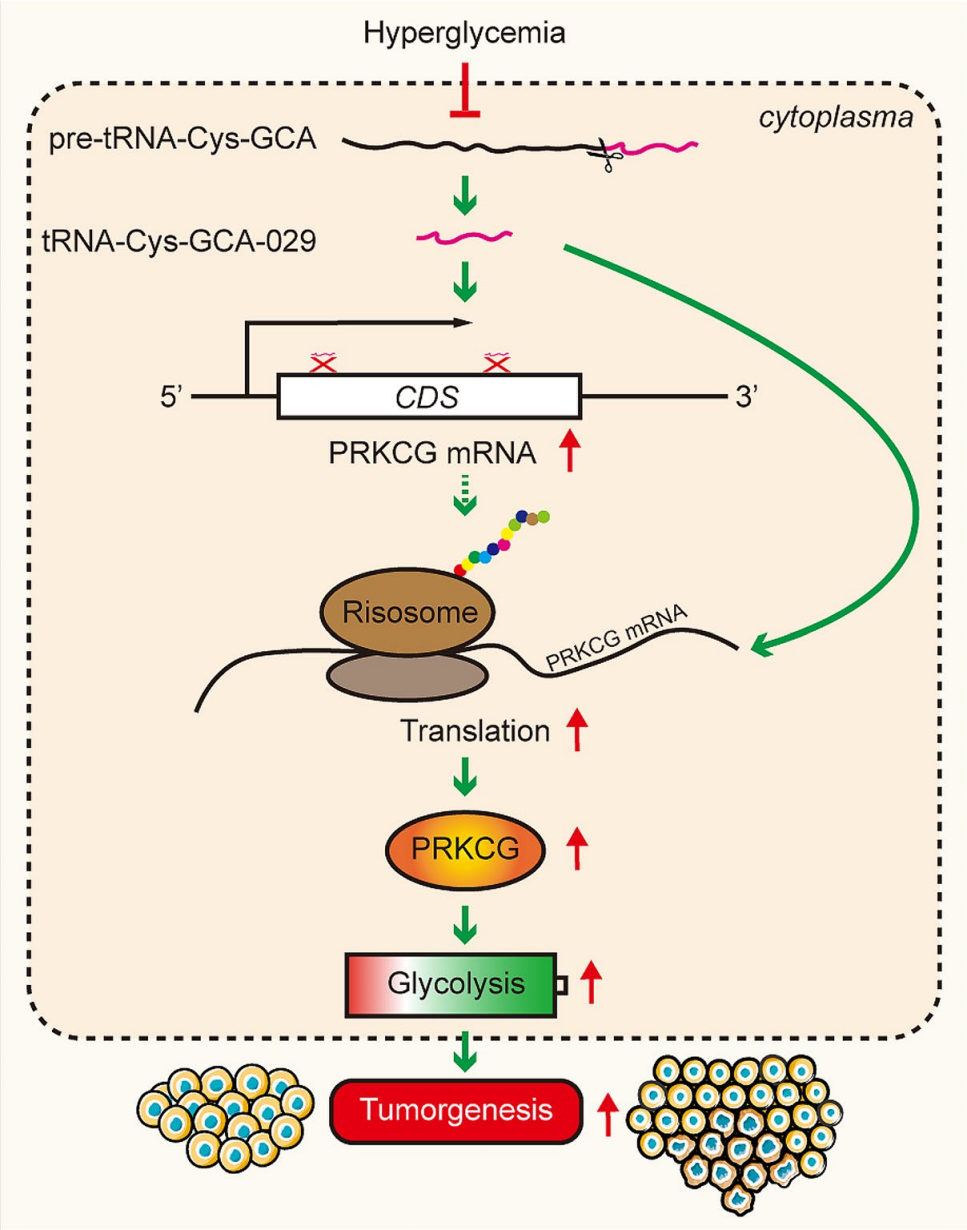

**Supplementary Information:**

The online version contains supplementary material available at 10.1186/s13058-024-01870-1.

## Introduction

Breast cancer (BC) and diabetes mellitus (DM) are both common diseases that have substantial impacts on human health globally. Particularly, BC has surpassed lung cancer as the most common cancer worldwide in 2020, with approximately 2.3 million new cases per year [1]. Moreover, there are more than 422 million people throughout the world suffering from DM, 85–95% of whom have type-2 diabetes (T2DM) [2]. Over the past decades, increasing epidemiological evidence have indicated that DM is a major risk factor for BC [3–5]. For example, several meta-analyses have concluded that women with DM have a 20–28% increased risk for the occurrence of BC [4, 6]. Indeed, it has been documented that up to one-third of women have DM at the time of BC diagnosis [4, 7], resulting in a mixed phenotype of patients with co-existing BC and DM (BC-DM). There are also reports that BC-DM patients have up to 57% higher risk of mortality than BC subjects without DM (BC-no-DM) [8–10]. In support of these, clinical studies have indicated that anti-diabetic therapies such as metformin use are associated with improved prognosis of BC [11, 12]. Thus, there is strong evidence linking DM to BC development and progression. Nevertheless, few studies have explored the molecular mechanisms underlying the pathogenesis of BC-DM.

tRNA-derived fragments (tRFs) are an emerging class of small non-coding RNAs cleaved from precursor or mature transfer RNAs (tRNAs) [13, 14]. Accumulating reports have reported that tRFs are widely involved in many biological processes, such as gene expression, protein synthesis, and RNA processing [15, 16]. Dysregulation of tRFs has been associated with the initiation and development of human cancers, including BC [17]. For instance, tRNA^Leu^-derived tRF was found to suppress stem cell–like cells and metastasis in colorectal cancer by targeting the Notch ligand JAG2 [18]. tRF-21, which is derived from tRNA^GlyGCC^, was downregulated in pancreatic ductal adenocarcinoma (PDAC) and its inhibition promoted PDAC cell malignancy [19]. It has been found that tRFs generated from tRNA^Glu^, tRNA^Asp^, tRNA^Gly^, and tRNA^Tyr^ could bind to YBX1 and displace YBX1 from the 3’-UTRs of oncogenic mRNAs, leading to their degradation and thus suppressing breast cancer metastasis [20]. 5’-tRF^Cys^ is required for breast cancer metastasis by binding to metabolic transcripts Mthfd1l and Pafah1b1 to form a stabilizing ribonucleoprotein complex [21]. In our previous work, we found that As-tDR-007333 promoted non-small cell lung cancer (NSCLC) malignancy through regulating H3K4me1 and H3K27ac modifications and augmenting ELK4-mediated MED29 transcription [22]. We also showed that circulating As-tDR-007333 level was a potential diagnostic biomarker for NSCLC [22]. Importantly, we and others demonstrated that targeting tRFs with complementary oligonucleotides inhibited tumor growth with little observed toxicity [22, 23]. Overall, these findings suggest that tRFs are critical regulators of human cancers. However, whether tRFs may play a role in BC-DM is unknown.

In this study, we used tRF&tiRNA sequencing to investigate the expression profiles of tRFs in BC-DM tumor tissues. We identified that tRF-Cys-GCA-029 was downregulated in BC-DM and in hyperglycemia conditions. We revealed that inactivation of tRF-Cys-GCA-029 promoted the malignancy of BC cells in a glucose level-dependent manner. We further demonstrated that tRF-Cys-GCA-029 regulated the malignancy and glycolysis metabolism of BC cells through modifying the expression and translation of PRKCG gene. Our results indicate that tRF-Cys-GCA-029 is a BC-DM-associated tRF and highlight the potential of tRF-Cys-GCA-029 as a promising therapeutic target for BC-DM.

## Methods

### Clinical sample collection

The study was approved by the Clinical Research Ethics Committee of Shenzhen University Health Science Center (Approved No. 2,019,015). Written informed consent was obtained from all study patients. Tumor tissues of BC-DM and BC-no-DM patients were obtained from subjects who underwent surgical resection at Cancer Hospital of Harbin Medical University and Peking University Shenzhen Hospital. After surgical resection, tumor tissues were quickly frozen in liquid nitrogen and then stored at − 80 °C until further processing. All patients had a definite pathological diagnosis of breast cancer. No patients received radiotherapy or chemotherapy prior to surgery. The histological type, grade, and TNM-stage were classified according to the American Joint Committee on Cancer(AJCC)TNM staging system (7th edition) [24] Diabetes was defined as having fasting plasma glucose (FPG) level ≧ 7.0 mmol/l, according to the criteria of American Diabetes Association (ADA) [25]. Clinicopathological characteristics of patients were summarized in Table S1–2.

### tRF & tiRNA sequencing

Total RNA was extracted from tumor tissues using the TRIzol^®^ reagent (Invitrogen, MA, USA), according to the manufacturer’s protocol. To remove RNA modifications that affect small RNA-seq library construction, the RNA samples were first pretreated with the following reagents: 3’-aminoacyl (charged) deacylation to 3’-OH for 3’adaptor ligation, 3’-cP (2’,3’-cyclic phosphate) removal to 3’-OH for 3’adaptor ligation, 5’-OH (hydroxyl group) phosphorylation to 5’-P for 5’-adaptor ligation, m1A and m3C demethylation for efficient reverse transcription. Pretreated RNA sample was then subjected to tRF&tiRNA-seq library preparation. Library preparation procedures included: 3’ and 5-adapter ligation, cDNA synthesis, PCR amplification, and size selection of 134–160 bp PCR amplified fragments (corresponding to 14-40nt small RNAs). The completed libraries were sequenced on NextSeq system using NextSeq 500/550 V2 kit (Illumina) at Aksomics Inc. (Shanghai, China). Image analysis and base calling were performed using Solexa pipeline v1.8 (Off-Line Base Caller software, v1.8). Sequencing qualities were examined by FastQC and trimmed reads were aligned to the precursor and mature tRNA sequences in GtRNAdb database by bowtie software. The remaining reads were aligned to miRNA reference sequences with miRDeep2 database. The tRF & tiRNA expression levels were measured and normalized to the number of transcripts per million of total aligned tRNA reads (TPM). The differential expression of tRFs& tiRNAs between groups were compared by calculating the fold change for each tRF. Paired P-value < 0.05 was considered statistically significant.

### Cell culture and transfection

The human BC cell lines (MDA-MB-231, MCF-7, BT-549, and T-47D) were obtained from the Cell Bank of Chinese Academy of Biological Sciences (Shanghai, China). All cell lines were authenticated through short-tandem repeat (STR) DNA profiling. No contamination of mycoplasma was found in these cell lines. Among these cell lines, MDA-MB-231, MCF-7, and BT-549 cells were cultured in Dulbecco’s Modified Eagle’s medium (DMEM) (Gibco, Shanghai, China) supplemented with 10% (v/v) fetal bovine serum (FBS) (Gibco, Shanghai, China, Table S3). T-47D cells were incubated in RPMI 1640 (Gibco, Shanghai, China) containing 10% FBS. All cells were incubated in a humidified atmosphere of 5% CO_2_ at 37^o^C.

The tRF-Cys-GCA-029 mimic, tRF-Cys-GCA-029-inhibitor, and their corresponding negative controls (NC) were synthesized by Ribobio Co. (Guangzhou, China). Cells were cultured on 6-well plates to confluence for 24 h. The tRF mimic, inhibitor, and NCs were transfected into cells, respectively, using Lipofectamine 3000 reagent (Table S4) according to the manufacturer’s instructions.

### Quantitative real-time PCR (qRT-PCR)

Total RNA was isolated using the TRIzol reagents (Life Technologies, Shanghai China) according to the manufacturer’s protocol. 1 mg of total RNA was reverse transcribed into cDNA using the Takara cDNA kit (Takara Bio, Beijing, China). The transcript level of specific gene or tRF was amplified using the Takara qPCR kit and was normalized to U6. The primers were synthesized by Ribobio Co. (Guangzhou, China).

### Cell proliferation assay

Cell Counting Kit-8 (CCK-8, Dojindo, Shanghai, China) kit was used to examine cell proliferation rate according to the manufacturer’s instructions. Briefly, the cells were planted on 96-well plate with a density of 5 × 10^3^ cells/well. After incubation for 24, 48, 72, 96, 120 h,10 µl CCK-8 reagent and 100 µl fresh DMEM with 10% FBS were added, then cells were incubated at 37 ℃ for 1 h. The absorbance at 450 nm was measured by a microplate reader.

### Cell migration assay

Cell migration was determined by Falcon Transwell chambers (Life Sciences, Shanghai, China) following the manufacturer’s instructions. Briefly, transfected cells were placed on the upper surface of the transwell insert. After 16 h, the invasive cells in the lower chamber were collected and washed with PBS, and then fixed with methanol for 60 mins. The fixed samples were washed and stained with 1.0% crystal violet staining solution (Beyotime, Shanghai, China). The number of invasive cells were counted in five randomly selected microscope visions and photographed.

### Apoptosis assay

FITC Annexin V Apoptosis Detection Kit (BD Biosciences, CA, USA) was used to measure cell apoptosis, according to the manufactures’ protocols. In brief, BC cells transfected with tRF-Cys-GCA-029 mimic, inhibitor, or NC were stained with Annexin V-FITC and propidium iodide. The apoptotic rates of cells were analyzed using flow cytometer (Beckman Coulter, Inc., IN, USA) equipped with CytExpert software.

### Diabetes-breast cancer animal model

BalB/C mice were purchased from the Biocytogen Animal Center (Zhuhai, China). Mice were firstly acclimated to the housing facilities for 7 days and then fed with high fat diet (HFD, 1135DM-5, Boaigang, Beijing, China). After 12 days of HFD feeding, streptozotocin (STZ, once 50 mg/kg body weight/day for 3 days) was injected intraperitoneally to induce diabetes. The corresponding diet lasted for the entire experimental period (50 days). Glycemic levels were measured on whole blood obtained from the snipped mouse tail using a glucometer (Yuwell 550, Shanghai, China). Diabetes was confirmed by the presence of hyperglycemia (> 11.1 mmol). 4T1 cells (2.5 × 10^5^) were subcutaneously injected into the right flank of mice. When the tumors attained a size of 50 mm^3^, mice were randomly divided into 2 groups: control group (injected with5 nmol/10µl tRF-Cys-GCA-029 NC), tRF treatment group (injected with 5 nmol/10µl tRF-Cys-GCA-029 mimic). NC or tRF mimic were locally injected into the tumor mass once every 3 days, respectively (Fig. 4A). After implantation, mice were followed with repeated measurements of blood glucose levels and body weight. A caliper was used to measure the length and width of each tumor. The equation of volume = length × width^2^ was used to calculate tumor volumes. After two weeks treatment (a total of 6 injections), the animals were sacrificed, and the xenograft tumors were excised for tumor weight analyses. All animal study procedures were performed in accordance with the guidelines approved by the Institutional Animal Care and use Committee of Shenzhen University Medical School (Approved No. IACUC-202,300,060).

### RNA-seq

RNA sequencing was carried out to identify the target genes regulated by tRF-Cys-GCA-029. MDA-MB-231 cells were transfected with tRF-Cys-GCA-029 mimic or scrambled control for 48 h. Three biological replicates for each sample were included in this experiment. Thereafter, RNA was isolated from the treated MDA-MB-231 cells using the TRIzol (Invitrogen, Shanghai, China) reagents. The total RNA quantity and purity were analyzed with Bioanalyzer 2100 and RNA 6000 Nano LabChip Kit (Agilent, CA, USA). High-quality RNA samples with RIN number > 7.0 were used to construct sequencing library, and then performed the 2 × 150 bp paired-end sequencing (PE150) on an Illumina Novaseq™ 6000 (at LC-Bio Technology CO., Ltd., Hangzhou, China) following the vendor’s recommended protocol. We aligned reads of all samples to the reference genome database using the HISAT2 package (version 2.1.0), which initially removed a portion of the reads based on quality information and then mapped the reads to the reference genome. The mapped reads of each sample were assembled using StringTie software (version 1.3.3) with default parameters. Genes differentially expressed between sample groups were calculated using the DESeq2 software (Table S5). The genes with the parameter of false discovery rate (FDR) below 0.05 and absolute fold change ≥ 2 were considered differentially expressed genes.

### Quantification of L-lactate and pyruvate

For measurement of L-lactate and pyruvate levels, 1 × 10^6^ cells were seeded into each well of the six-well plate. Cells were transfected with tRF-Cys-GCA-029 mimic, tRF-Cys-GCA-029-inhibitor, and NCs, respectively. L-lactate and pyruvate levels in the supernatants of cell culture media were quantified using the L-lactate assay kit and the pyruvate assay kit (Jiancheng Bioengineering Institute, Nanjing, China) according to the manufacturer’s instructions. The sample absorbance (530 nm for L-lactate and 505 nm for pyruvate) was detected using the Synergy HTX Multi-Mode Microplate Reader (BioTek, Guangzhou, China). All experiments were performed in triplicate.

### Extracellular Acidification Rate (ECAR)

The extracellular acidification rate (ECAR) of cells were assessed using the Seahorse XFe24 Flux Analyzer (Seahorse Bioscience, Agilent). The glycolytic stress test kit (Seahorse) was used for ECAR detection. Briefly, the transfected MDA-MB-231 cells (1 × 10^5^ cells/well) were seeded in the 24-well XF Seahorse incubation microplate as the protocol indicated. Cells were cultured at 37 °C overnight in XF base medium (pH 7.4) for adhesion. After baseline measurements, glucose (10 mM), glutamine (1 mM), 2-DG (50 mM) and oligomycin (1 µM) were added sequentially into each well at indicated time points. Data of ECAR were analyzed by Seahorse XF Glycolysis Stress Test Report Generator package.

### Western blot analysis

Cellular proteins were extracted with cell lysis buffer (Promega, Madison, WI, USA). Thirty microgram of protein extraction was separated on an 10% sodium dodecyl sulfate-polyacrylamide Gel electrophoresis (SDS-PAGE) gel and then transferred to polyvinylidene fluoride (PVDF) membranes. After blockage by 5% Bovine serum albumin (BSA) at room temperature for 1 h, the membranes were incubated with primary antibodies (anti-PRKCG, 1:2000 dilution, Proteintech, anti-β-actin, 1:1000 dilution, Abcam) at 4 °C overnight. Then, blots were incubated at room temperature for 90 min with horseradish peroxidase (HRP) conjugated beta-actin secondary antibodies (diluted 1:5000, ZSGB-BIOZS). The bands were scanned and visualized by the GS700 imaging densitometer (Bio-Rad Laboratories) and analyzed by Image Studio software.

### Ribosome profiling

Polysome profiling was performed as previously reported [26]. In brief, BC cells were pretreated with cycloheximide (CHX,100 µg/ml) at 37 °C for 15 min. Cells were collected and lysed on ice with lysis buffer (20mM Tris-HCl (pH 7.5),50mM KCl,10mM MgCl2, 1mM DTT,100mM CHX,200 µg/ml Heparin,1% Triton X-100) for 10 min. After centrifuging at 13,000 g for 10 min at 4 °C, the lysate was loaded onto a 10-50% sucrose gradient and further ultracentrifuged at 4 °C for 4 h at 36,000 rpm. Samples were subjected to fractionate using the Gradient Station (BioComp Instruments, Fredericton, Canada) and monitored at 254 nm. Thirteen fractions of equal volume were collected and their absorbances were measured. The 1–6 fractions were pooled together as light fraction (monosome) and the fractions 7–13 were pooled as the heavy weight fraction (polysome). The fractions were lysed in TRIzol (Invitrogen™) and isolated RNA was used for qRT-qPCR analysis.

### Luciferase reporter assays

The PRKCG wild-type (WT) and mutant-type (MT) sequences were cloned into pmirGLO-basic vector. HEK-293T cells were cotransfected with luciferase reporters, either WT or MT, in combination with tRF-Cys-GCA-029 mimic or NC, using Lipofectamine 2000 reagents (Invitrogen, 11668-027). The luciferase activity was measured by Dual-Luciferase Reporter Assay System (Promega, E1910). And then the firefly luciferase activity values were normalized to the Renilla luciferase activity values that reflect expression efficiency.

### Bioinformatics and statistical analyses

PRKCG mRNA sequence was downloaded from the NCBI RefSeq database (https://www.ncbi.nlm.nih.gov/gene/; NCBI transcription ID: NM_0013163.29.2). The putative tRF-Cys-GCA-029 binding sites in the PRKCG genome sequence were predicted on the basis of 20 kcal/mol of minimal free energy threshold using two independent bioinformatics tools, Targetscan and miRanda (Table S5).

Data are expressed as the means ± SD from at least three independent experiments. Statistical analyses were performed using Graph Pad Prism 8 and (Graph Pad, USA) and R program. Comparison between groups was conducted using Student’s t-test (for parametric data) or the Mann–Whitney test (for non-parametric data). All tests were two tails and P-value < 0.05 was considered statistically significant.

## Results

### tRFs are differentially expressed between BC-DM tumor tissues and BC-no-DM tumor tissues

To investigate the expression profiles of tRFs in BC-DM, tRF&tiRNA sequencing was implemented in 6 tumor tissues from BC-DM patients and 6 tumor tissues from BC-no-DM subjects (Table S1). The volcano plotting and principal component analysis (PCA) showed systematic variation in tRF expression between BC-DM and BD-no-DM tumor tissues (Fig. S1A, B). Ven diagram analysis revealed that there were 51 and 27 unique tRFs for BC-DM and BC-no-DM, respectively (Fig. S1C). The tRF-5c, formed by cleavage at the D- and anticodon stems, was the most abundant tRF subtype in both the BC-DM and BC-no-DM groups (Fig. S1D, E). The stacked bar analysis showed that different subtypes of tRFs have different length range. For example, most tRF-5c were 28–32nt in length, while the length range of tRF-1s was larger (14-27nt) (Fig. S1F). tRNA isodecoder analysis showed that tRF-5c was mainly originated from Glu-TTC, Glu-TTC, Gly–CCC, Gly-GCC, Gly-TCC, His-GTG, Lys-CTT, and Val-CAC (Fig. S1G). As can be seen from Fig. S1H, the tRF subtypes were more abundant in lengths between 20-24nt and 30-34nt subtypes for both BC-DM and BC-no-DM samples, but the length distribution profiles were different between BC-DM and BC-no-DM tumor tissues. Collectively, these data indicate that the expression profiles of tRFs in BC-DM were different from that in BC-no-DM. Given that tRF expression profiles are known to be tissue- and cell-type specific in human cancers [27, 28], the observed differentially expressed tRFs in BC-DM suggest that tRFs may be involved in the pathogenesis of BC-DM.

### tRF-Cys-GCA-029 is downregulated in BC-DM and under hyperglycemia conditions

**Fig. 1:**
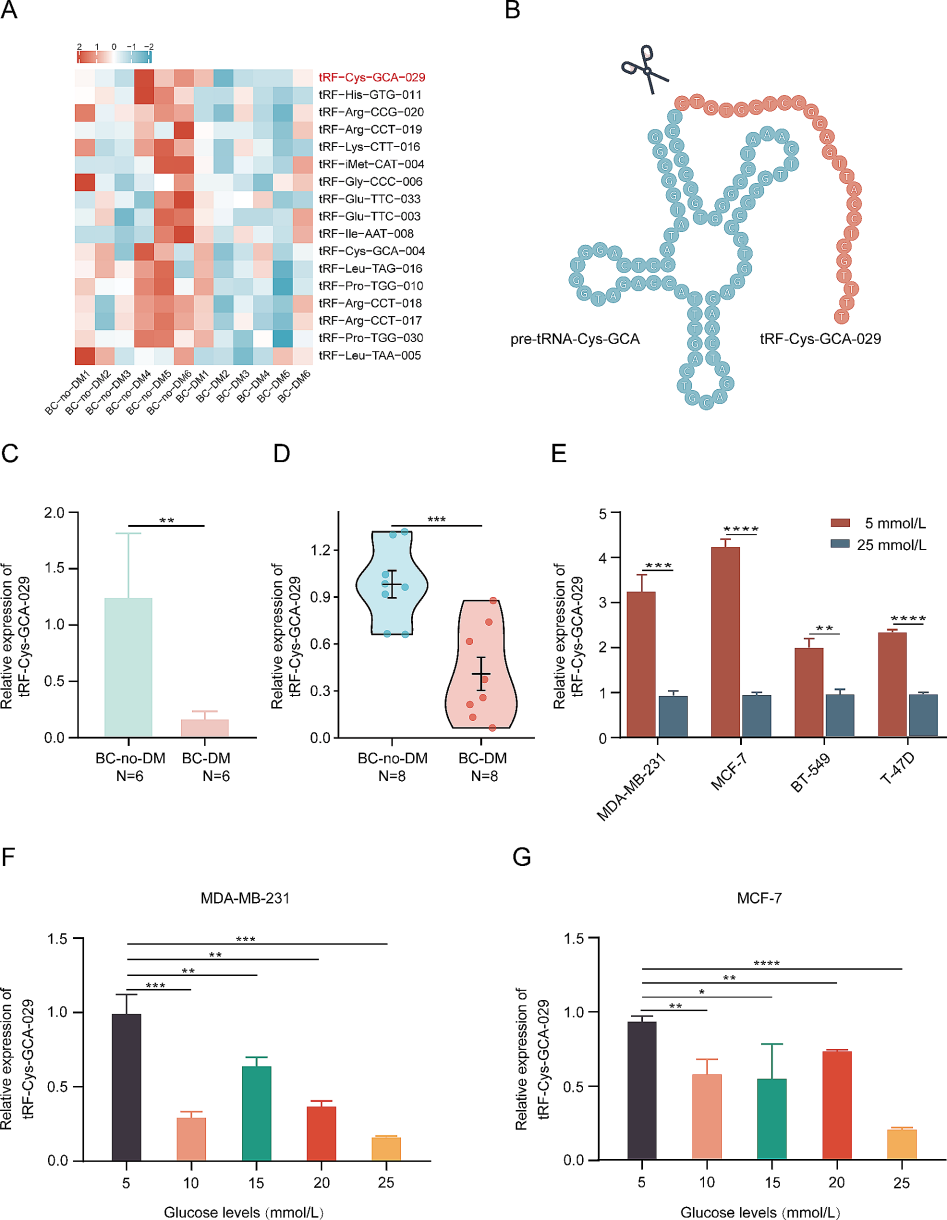
tRF-Cys-GCA-029 is down-regulated in BC-DM and under hyperglycemia conditions. (**A**) Heatmap of top 20 differentially expressed tRFs between BC-DM tumor tissues and BC-no-DM tumor tissues, indicating that tRF-Cys-GCA-029 is the most significantly down-regulated tRF in tumor tissues of BC-DM. (**B**) Schematic illustration of the secondary structures of tRF-Cys, revealing the nucleotides that give rise to tRF-Cys-GCA-029 colored in orange. (**C**) qRT-PCR assay confirms the lower abundance of tRF-Cys-GCA-029 in BC-DM tissues compared with that in BC-no-DM tissues (Harbin samples). (**D**) tRF-Cys-GCA-029 expression levels in tumor tissues of BC-DM are significantly lower than that in BC-no-DM (Shenzhen samples). (**E**) High glucose level inhibits tRF-Cys-GCA-029 expression in BC cells. (**F**) Low glucose concentration promotes tRF-Cys-GCA-029 expression in MDA-MB-231 cells. (**G**) tRF-Cys-GCA-029 expression levels are increased under lower glucose levels in MCF-7 cells. *P < 0.05, ** *P* < 0.01, *** *P* < 0.001

Comparison of tRF expression profiles between BC-DM and BC-no-DM revealed 68 differentially expressed (fold change≧∣2.0∣, *P* < 0.05) tRFs in BC-DM tumor tissues, of which 36 were up-regulated and 32 were down-regulated. Hierarchical clustering heatmap plotting suggested that tRF-Cys-GCA-029 was the top downregulated tRF in BC-DM (Fig. 1A and B, Fig. S2). Further analysis with qRT-PCR assay confirmed that the expression of tRF-Cys-GCA-029 was significantly lower in BC-DM tissues than that in BC-no-DM tissues in both sample set 1 and sample set 2 (Fig. 1C and D). To explore whether the expression of tRF-Cys-GCA-029 was associated with glucose levels, we cultured MDA-MB-231 and MCF-7 cells at normal physical levels of glucose (5 mmol/L) or at different concentration of hyperglycemia (10, 15, 20, 25 mmol/L), respectively. Then we measured the expression levels of tRF-Cys-GCA-029 in BC cells under different glucose conditions. The results showed that tRF-Cys-GCA-029 expression was increased under euglycemia conditions but decreased under hyperglycemia cultures in all types of BC cell lines studied. (Fig. 1E). Interestingly, there was a trend of higher tRF-Cys-GCA-029 expression in response with lower glucose levels, with lowest level of tRF-Cys-GCA-029 expression seen in culture media with 25 mmol/L glucose (Fig. 1F and G). In summary, these data suggest that tRF-Cys-GCA-029 is downregulated under diabetic and hyperglycemia conditions, and may play an oncogenic role in the BC-DM.

### Low expression of tRF-Cys-GCA-029 promotes proliferation and migration of BC cells in a glucose level-dependent manner

**Fig. 2:**
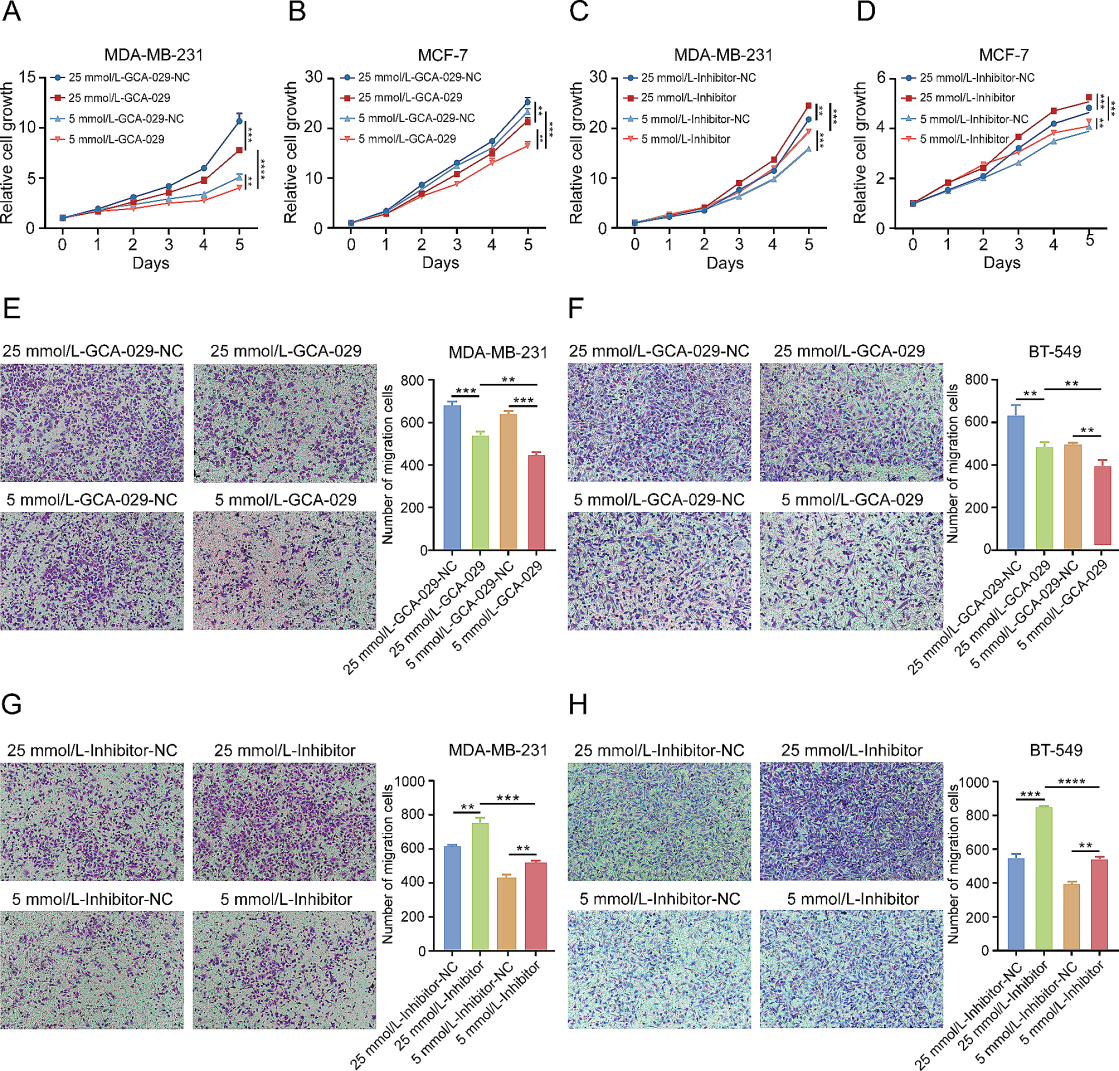
tRF-Cys-GCA-029 regulates BC cell proliferation and migration in a glucose level-dependent manner. (**A**) Overexpression of tRF-Cys-GCA-029 inhibits cell proliferation in a glucose-level dependent manner in MDA-MB-231 cells. (**B**) Upregulation of tRF-Cys-GCA-029 suppresses cell viability in a glucose-level dependent fashion. (**C**) The promoting effect of tRF-Cys-GCA-029 downregulation on cell proliferation is stronger under hyperglycemic conditions than that under euglycemic conditions in MDA-MB-231 cells. (**D**) The enhancing effect of tRF-Cys-GCA-029 silencing on cell proliferation is greater under hyperglycemic conditions than that under euglycemic conditions in MCF-7 cells. (**E**) Up-regulation of tRF-Cys-GCA-029 suppresses migration capacity of MDA-MB-231 cells in a glucose level dependent manner. (**F**) Hyperglycemia facilitates theinhibitive effects of tRF-Cys-GCA-029 on migration ability of BT-549 cells in a glucose concentration dependent fashion. (**G**) Higher glucose levels increase the promoting effect of tRF-Cys-GCA-029 knockdown on cell migration in MDA-MB-231 cells. (**H**) Downregulation of tRF-Cys-GCA-029 enhances the migration ability of BT-549 cells in a glucose-dependent manner. * *P* < 0.05, ** *P* < 0.01, *** *P* < 0.001

To examine the biological roles of tRF-Cys-GCA-029 in BC cells and to explore the influence of glucose level on the biological functions of tRF-Cys-GCA-029, we transfected synthesized tRF-Cys-GCA-029 mimic and tRF-Cys-GCA-029-inhibitor into BC cells to perform gain- and loss- of function experiments. The sequences of tRF-Cys-GCA-029 mimic and inhibitor are shown in Table S3. The transfection efficiencies were tested by qRT-PCR (Fig. S3). Under both normal glucose and hyperglycemia conditions, BC cell proliferation rate was significantly suppressed by overexpression of tRF-Cys-GCA-029 in comparison with the control cells (Figure 2A and B). In contrast, tRF-Cys-GCA-029-inhibitor enhanced the proliferation rate of BC cells (Fig. 2C and D). Notably, the impacts of tRF-Cys-GCA-029 on cell proliferation were stronger in hyperglycemia conditions than that in normal glucose culture conditions. These data suggest that the effects of tRF-Cys-GCA-029 on BC cell proliferation are dependent on glycemic levels. In consistent with this, results from migration assay revealed that tRF-Cys-GCA-029-overexpression suppressed migration capacity of BC cells while tRF-Cys-GCA-029-inhibitor increased the migration rates of BC cells (Fig. 2E-H). Similarly, the effects of hyperglycemia on cell migration capacity were significantly stronger than that of normal glucose treatment. However, the effects of tRF-Cys-GCA-029 or its inhibitor on apoptosis rate of BC cells were moderate with limited statistical power (Fig. S4). Taken together, these findings indicate that the effects of tRF-Cys-GCA-029 on BC cell proliferation and migration are dependent on extracellular glucose levels.

### Silencing of tRF-Cys-GCA-029 enhances glycolysis metabolism in BC cells

**Fig. 3:**
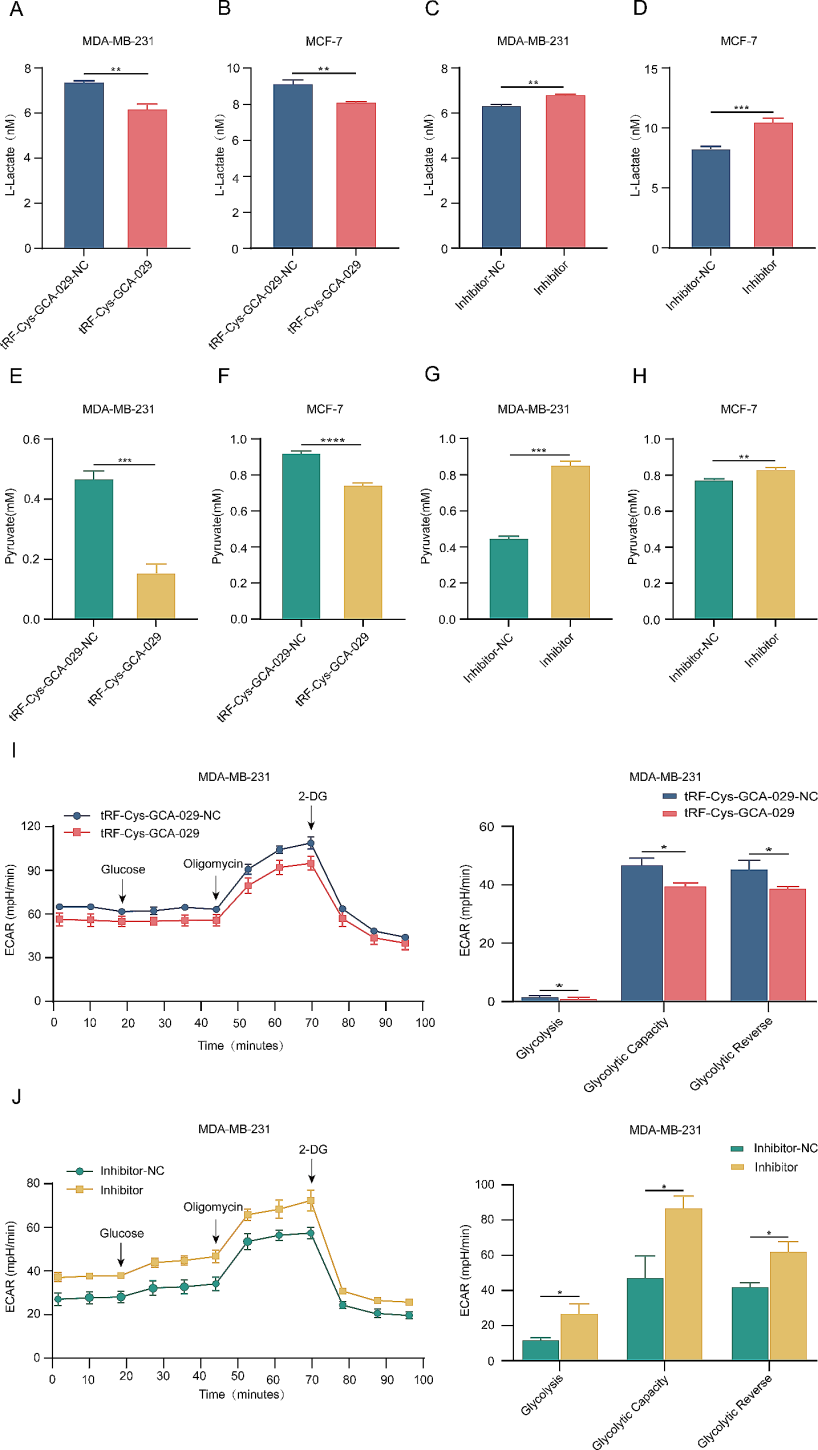
Silencing of tRF-Cys-GCA-029 enhances glycolysis metabolism in BC cells. (**A**) Overexpression of tRF-Cys-GCA-029 inhibits the release of L-lactate from MDA-MB-231 cells. (**B**) Higher expression of tRF-Cys-GCA-029 suppresses the production of L-lactate in MCF-7 cells. (**C**) Inhibition of tRF-Cys-GCA-029 increases the generation of L-lactate from MDA-MB-231 cells. (**D**) Silencing of tRF-Cys-GCA-029 promotes L-lactate production in MCF-7 cells. (**E**) Overexpression of tRF-Cys-GCA-029 suupresses the release of pyruvate from MDA-MB-231 cells. (**F**) Upregulation of tRF-Cys-GCA-029 represses the production of pyruvate in MCF-7 cells. (**G**) Down-regulation of tRF-Cys-GCA-029 increases the generation of pyruvate from MDA-MB-231 cells. (**H**) Knockdown of tRF-Cys-GCA-029increases pyruvate production in MCF-7 cells. (**I**) ECAR analysis shows that upregulation of tRF-Cys-GCA-029 inhibits the glycolytic capacity of MDA-MB-231 cells. (**J**) ECAR experiment indicates that downregulation of tRF-Cys-GCA-029 enhances glycolytic level of MDA-MB-231 cells. * *P* < 0.05, ** *P* < 0.01, *** *P* < 0.001

To further assess the biological function of tRF-Cys-GCA-029 in BC, we predicted the potential target genes of tRF-Cys-GCA-029 using the TargetScan and the miRanda databases. Then we performed GO and KEGG analyses to assess the potential biological functions of these predicted target genes. The GO analysis suggested that the target genes of tRF-Cys-GCA-029 were enriched in biological processes (BP) related to metabolism terms including primary metabolic process and regulation of cell metabolism (Fig. S5). Considering that glycolysis reprogramming is the most noteworthy metabolic process in tumor [29], we speculated that tRF-Cys-GCA-029 may take part in glycolysis metabolism in BC cells. Since in glycolysis process, glucose is mostly degenerated to pyruvic acid and then transformed to lactate, we focused our investigation on the measurement of glycolysis final products, pyruvate and lactate, as indicators of glycolysis. Lactate assay showed that over-expressed tRF-Cys-GCA-029 inhibited L-lactate production, while knockdown of tRF-Cys-GCA-029 increased L-lactate generation from BC cells (Fig. 3A-D). In agreement with lactate production, the pyruvate analysis demonstrated that tRF-Cys-GCA-029-upregulation suppressed but tRF-Cys-GCA-029-inhibition promoted pyruvate generation from BC cells (Fig. 3E-H). Furthermore, ECAR analysis in living BC cells confirmed that tRF-Cys-GCA-029 overexpression suppressed glycolytic level of BC cells, while knockdown of tRF-Cys-GCA-029 increased the glycolytic capacity (Fig. 3I and J). Thus, we conclude that tRF-Cys-GCA-029 downregulation facilitates glycolysis metabolism of BC cells.

### tRF-Cys-GCA-029 inhibits BC tumor growth in diabetic mice

**Fig. 4:**
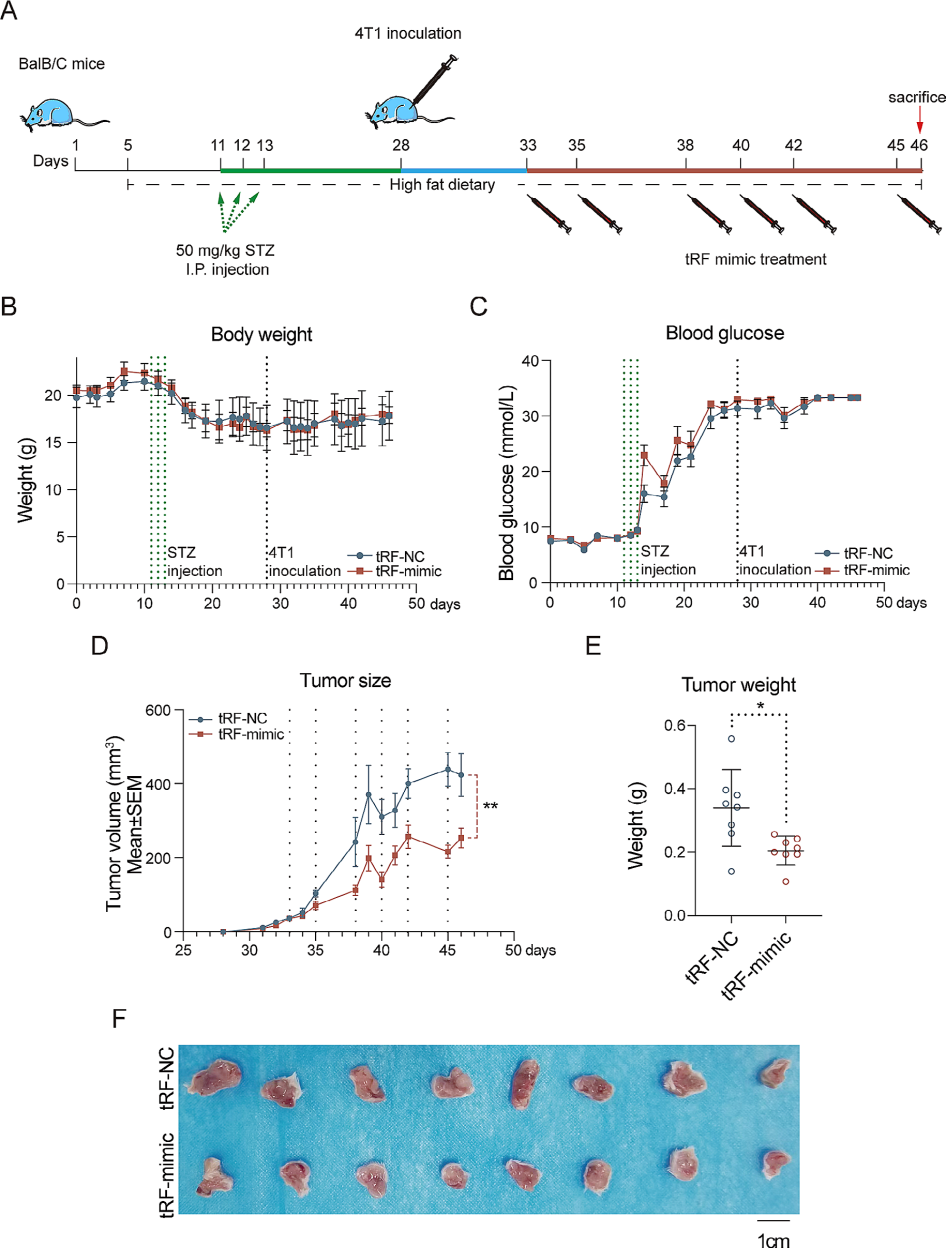
tRF-Cys-GCA-029 inhibits BC tumor growth under diabetic conditions in vivo. (**A**) Schematic overview and timeframe of the animal experiments. (**B**) Dynamic body weight changes of mice during the experiment. (**C**) Blood glucose concentrations in tRF-Cys-GCA-029 treatment group and controls during the entire experiment period. (**D**) Tumor growth curves show that tRF-Cys-GCA-029 administration suppresses tumor growth compared with that of NC group. (**E**) The mean xenograft tumor weight in tRF-Cys-GCA-029 treatment group is significantly lower than that of NC group. (**F**) Representative images of xenograft tumors in mice with or without tRF-Cys-GCA-029 mimic treatment. * *P* < 0.05, ** *P* < 0.01, *** *P* < 0.001

To validate the in vitro effects of tRF-Cys-GCA-029 on the malignancy of BC cells and the influence of hyperglycemia on the functions of tRF-Cys-GCA-029, we sought to investigate whether administration of tRF-Cys-GCA-029 may have any effect on BC in diabetic mice in vivo. We established STZ-induced diabetes model in obese BAIB/C mice (Fig. 4A). We maintained the mice with hyperglycemia for the entire experimental period (Fig. 4C). Then we constructed xenograft tumor models in these diabetic mice. The results showed that diabetic mice receiving tRF-Cys-GCA-029 mimic treatment had a significantly smaller tumor volume than that in NC group (Fig. 4D). Moreover, the mean tumor weight in the tRF-Cys-GCA-029-mimic group was significantly lower than that of mice without tRF-Cys-GCA-029 administration (Fig. 4E and F). But no differences in body weight were observed between experimental groups (Fig. 4B). Thus, our findings indicate that, under diabetic conditions, tRF-Cys-GCA-029 inhibits BC tumor growth in vivo.

### PRKCG is a direct target of tRF-Cys-GCA-029

**Fig. 5:**
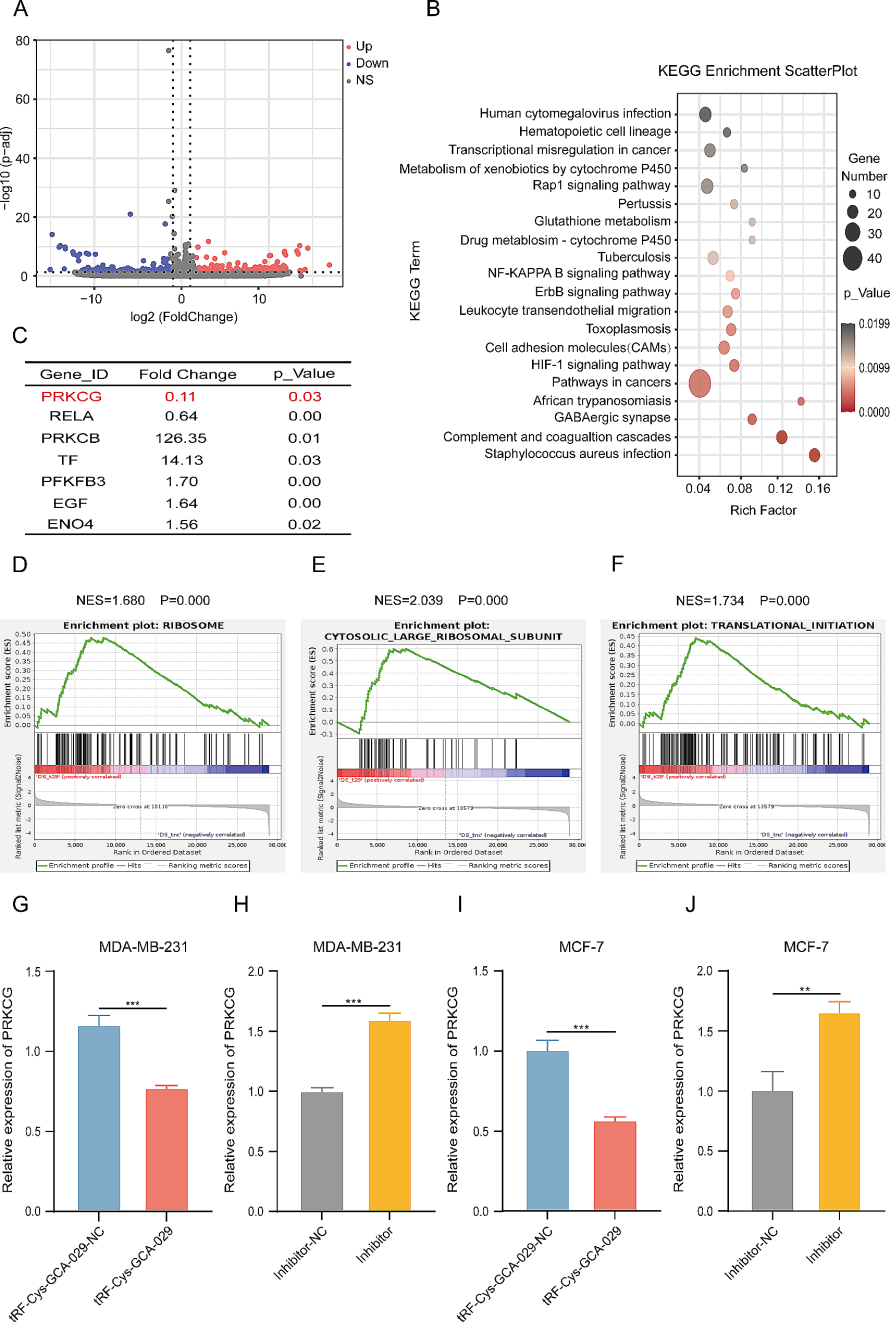
PRKCG is a direct target of tRF-Cys-GCA-029. (**A**) Volcano plot reveals that tRF-Cys-GCA-029 induces differentially expressed genes (DEG) in BC cells. (**B**) KEGG analysis shows that tRF-Cys-GCA-029-regulated DEGs are enriched in HIF-1α pathway and other metabolism-related pathways in MDA-MB-231 cells. (**C**) RNA-seq data shows that tRF-Cys-GCA-029 suppresses the expression of HIF-1α pathway gene PRKCG in MDA-MB-231 cells. (**D**,** E**,** F**) GSEA shows that tRF-Cys-GCA-029-regulated genes are enriched in ribosome, translational initiation, and larger ribosome subunit pathways. (**G**) qRT-PCR assay verifies that overexpression of tRF-Cys-GCA-029 suppresses the expression of PRKCG in MDA-MB-231 cells. (**H**) Knockdown of tRF-Cys-GCA-029 increases the expression levels of PRKCG in MDA-MB-231 cells. (**I**) Upregulation of tRF-Cys-GCA-029 inhibits the expression of PRKCG in MCF-7 cells. (**J**) Inhibition of tRF-Cys-GCA-029 promotes PRKCG expression in MCF-7 cells. * *P* < 0.05, ** *P* < 0.01, *** *P* < 0.001

To identify potential target gene of tRF-Cys-GCA-029, RNA-sequencing was performed in BC cells overexpressing tRF-Cys-GCA-029 and in BC cell with NC vector. As expected, tRF-Cys-GCA-029 induced a number of differentially expressed genes (DEG) (< 0.5-fold or > 2-fold, *P* < 0.05) between tRF-Cys-GCA-029-overexpressing BC cells and NC cells (Fig. 5A). KEGG analysis suggested that these DEGs were enriched in HIF-1α pathway, a key pathway involving in cellular glycolysis metabolism (Fig. 5B). Among these genes, PRKCG was one of the top outlier genes downregulated in tRF-Cys-GCA-029-overexpressing BC cells (Fig. 5C). Gene set enrichment analysis (GSEA) showed that tRF-Cys-GCA-029-regulated genes were enriched in ribosome content and translation initiation pathways (Fig. 5D-F), suggesting that tRF-Cys-GCA-029 may be involved in the translation activities of BC cells. The regulatory effects of tRF-Cys-GCA-029 on PRKCG expression was validated by qRT-PCR assay in both MDA-MB-231 and MCF-7 cells. It was observed that upregulation of tRF-Cys-GCA-029 suppressed the expression of PRKCG gene (Fig. 5G and I). Conversely, knockdown of tRF-Cys-GCA-029 upregulated PRKCG gene expression (Fig. 5H and J). Taken together, these results suggest that PRKCG is likely a target gene of tRF-Cys-GCA-029 in BC cells.

### PRKCG promotes the malignancy and glycolysis of BC cells

**Fig. 6:**
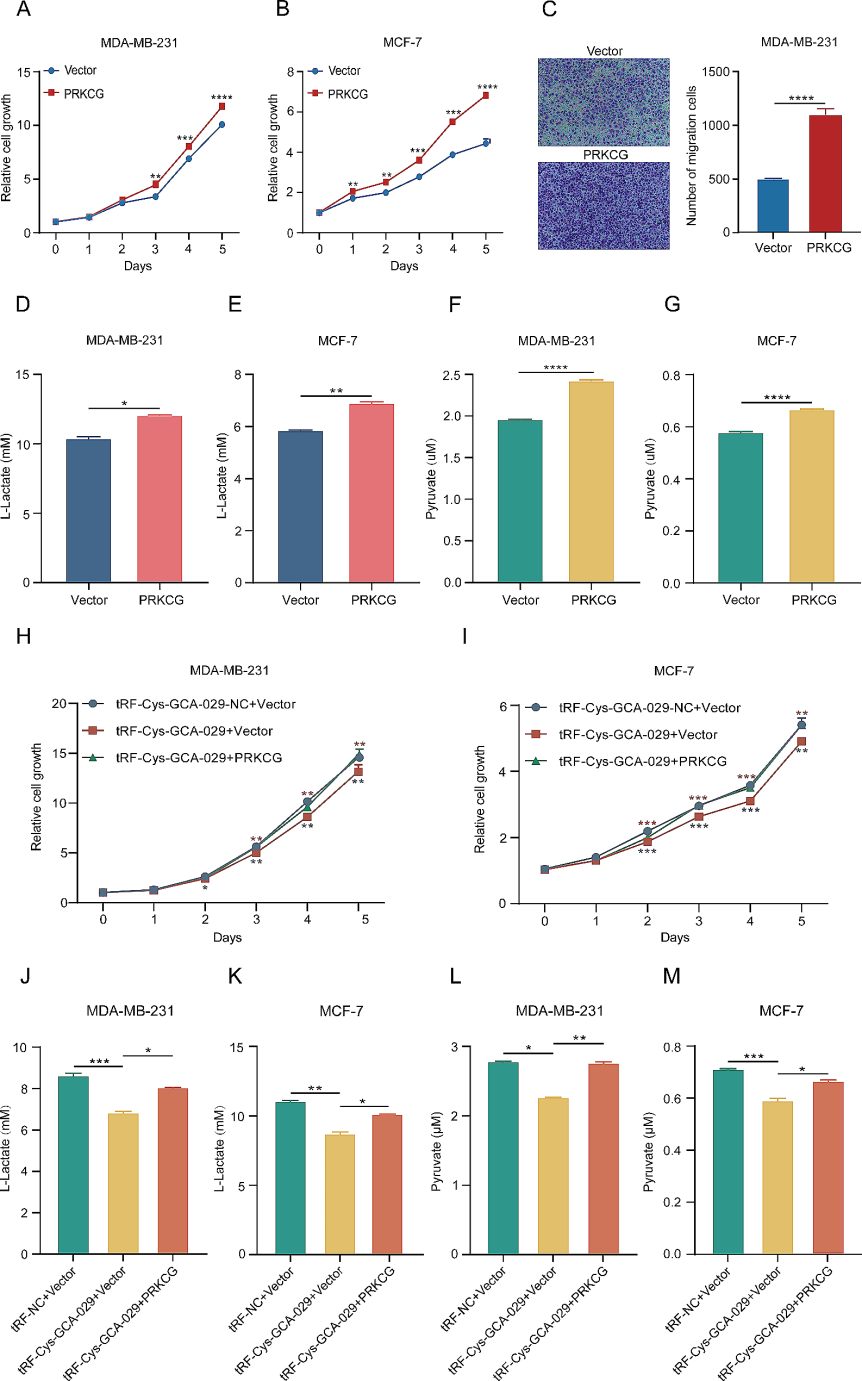
PRKCG is required for the tumor inhibiting and glycolysis reprograming abilities of tRF-Cys-GCA-029. (**A**) Overexpression of PRKCG promotes cell proliferation of MDA-MB-231 cells. (**B**) Upregulation of PRKCG increases cell proliferation of MCF-7 cells. (**C**) PRKCG enhances migration capacity of MDA-MB-231 cells. (**D**) Higher expression of PRKCG reduces L-lactate production in MDA-MB-231 cells. (**E**) Upregulation of PRKCG increases L-lactate release from MCF-7 cells. (**F**) Overexpression of PRKCG promotes pyruvate production in MDA-MB-231 cells. (**G**) Increased expression of PRKCG enhances pyruvate generation from MCF-7 cells. (**H**) Co-transfection of PRKCG and tRF-Cys-GCA-029 reverses the inhibitive effect of tRF-Cys-GCA-029 on cell proliferation of MDA-MB-231 cells. (**I**) The influence of tRF-Cys-GCA-029 on cell proliferation is rescued by PRKCG overexpression in MCF-7 cells. (**J**) Co-transfection of PRKCG and tRF-Cys-GCA-029 partly abolishes the effect of tRF-Cys-GCA-029 on L-lactate production in MDA-MB-231 cells. (**K**) The effect of tRF-Cys-GCA-029 on L-lactate generation is ameliorated by PRKCG in MCF-7 cells. (**L**) PRKCG reduces the inhibitive effect of tRF-Cys-GCA-029 on pyruvate production in MDA-MB-231 cells. (**M**) PRKCG reverses the suppressive effect of tRF-Cys-GCA-029 on pyruvate production in MCF-7 cells. * *P* < 0.05, ** *P* < 0.01, *** *P* < 0.001

To investigate whether tRF-Cys-GCA-029-regulated PRKCG may play a role in BC cell malignancy and glycolysis metabolism, we synthesized PRKCG-overexpressing plasmid and then transfected it into MDA-MB-231 and MCF-7 cells to construct gain-of-function cell models (Fig. S6). CCK8 assay showed that overexpression of PRKCG promoted BC cell proliferation (Fig. 6A, B). Cell migration assays also revealed that up-regulation of PRKCG significantly increased the migration capacity of BC cells (Fig. 6C). Moreover, overexpression of PRKCG significantly increased the production of L-lactate from BC cells (Fig. 6D, E). Similarly, upregulation of PRKCG enhanced the release of pyruvate from both MDA-MB-231 and MCF-7 cells (Fig. 6F and G). Collectively, these data indicate that PRKCG is an oncogene that promotes proliferation and glycolysis metabolism in BC cells.

### PRKCG is required for the tumor inhibiting and glycolysis reprograming activities of tRF-Cys-GCA-029

To examine whether the biological functions of tRF-Cys-GCA-029 on BC cell malignancy and glycolysis metabolism were mediated by PRKCG, a series of rescue experiments were conducted by co-transfecting tRF-Cys-GCA-029 mimic and PRKCG plasmidinto BC cells. The results showed that overexpression of PRKCG could markedly reverse the inhibiting effects of tRF-Cys-GCA-029 mimic on proliferation of MDA-MB-231 cells (Fig. 6H). Consistently, overexpression of PRKCG attenuated the suppressive roles of tRF-Cys-GCA-029 in MCF-7 cell proliferation (Fig. 6I). Moreover, L-lactate assay demonstrated that PRKCG overexpression evidently alleviated the effects of tRF-Cys-GCA-029 on L-lactate production in BC cells (Fig. 6J and K). Compared with NC cells, overexpression of PRKCG could counteract the repressing effects of tRF-Cys-GCA-029 on pyruvate production from BC cells (Fig. 6L and M). In summary, the above results provide evidence that tRF-Cys-GCA-029 depends on interaction with PRKCG to regulate cell proliferation and glycolysis metabolism in BC cells.

### tRF-Cys-GCA-029 binds to the coding sequence (CDS) of PRKCG to regulate its transcription

**Fig. 7:**
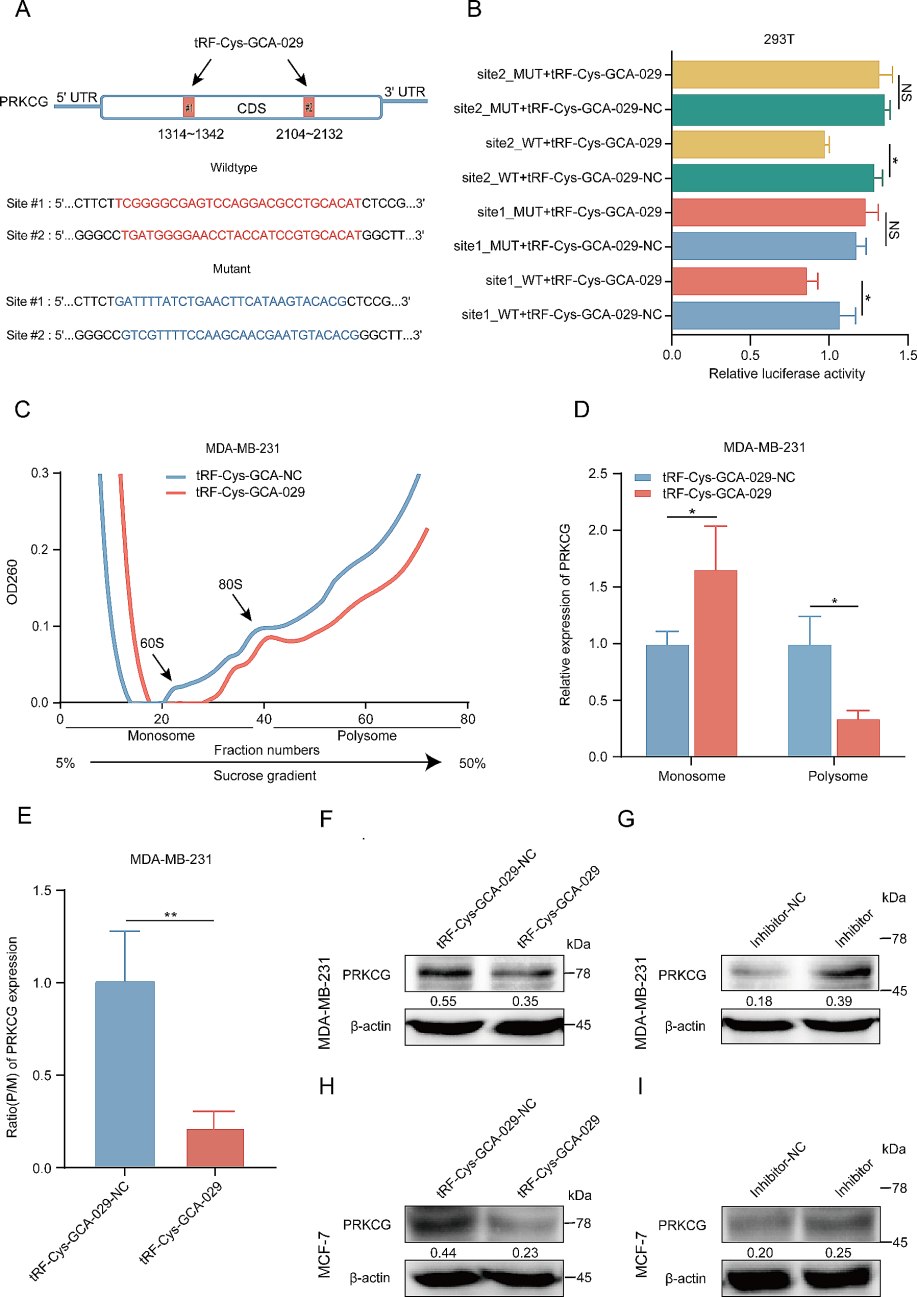
tRF-Cys-GCA-029 regulates PRKCG expression and translation in BC cells. (**A**) Schematic illustration of wild type and mutant sequences of two putative binding sites of tRF-Cys-GCA-029 in the CDS of PRKCG mRNA. (**B**) Luciferase activity of WT, but not the MT, PRKCG CDS is significantly decreased by tRF-Cys-GCA-029 transfection in HEK-293T cells. WT: wild-type sequence; MT: mutated sequence. (**C**) Polysome profiles between tRF-Cys-GCA-029 overexpression HEK-293T cells and NC cells. (**D**) Upregulation of tRF-Cys-GCA-029 increases the abundance of PRKCG mRNA in monosome but reduces PRKCG expression level in polysomes. (**E**) Overexpression of tRF-Cys-GCA-029 decreases the polysome to monosome ratio of PRKCG mRNA expression, indicating a reduction of PRKCG translation. (**F**) Upregulation of tRF-Cys-GCA-029 suppresses PRKCG protein expression in MDA-MB-231 cells. (**G**) Knockdown of tRF-Cys-GCA-029 increases PRKCG protein expression in MDA-MB-231 cells. (**H**) Over-expression of tRF-Cys-GCA-029 inhibits PRKCG protein expression in MCF-7 cells. (**I)** Inhibition of tRF-Cys-GCA-029 enhances PRKCG protein expression in MCF-7 cells. * *P* < 0.05, ** *P* < 0.01, *** *P* < 0.001

To elucidate the biological significance and its underlying mechanisms of tRF-Cys-GCA-029 downregulation on PRKCG, we analyzed the PRKCG gene sequence using the miRanda and TargetScan programs. Bioinformatic analyses revealed that there were 2 possible binding sites in the coding sequence (CDS) of PRKCG gene for tRF-Cys-GCA-029 (Fig. 7A). To investigate whether tRF-Cys-GCA-029 may regulate PRKCG expression, we constructed luciferase-reporter plasmids containing the wild type and mutant PRKCG CDS sequences, respectively. The wild type or mutant constructs were co-transfected with tRF-Cys-GCA-029 mimic into HEK-293T cells, respectively. Our results showed that the reporter vectors with wildtype targeting sequence of PRKCG caused a significant decrease in luciferase activity in cells transfected with tRF-Cys-GCA-029 mimic. Whereas reporter plasmids with mutant sequences of PRKCG produced no change in luciferase activity (Fig. 7B). These results suggest that tRF-Cys-GCA-029 may inhibit the transcription of PRKCG by directly targeting its CDS sequence.

### tRF-Cys-GCA-029 modifies PRKCG mRNA translation by regulating its expression in the polysomes

Since the expression of PRKCG protein was regulated by tRF-Cys-GCA-029, and tRF-Cys-GCA-029-modified genes were enriched in ribosome and translational initiation pathways, we hypothesized that tRF-Cys-GCA-029 may affect PRKCG protein expression by modulating its translation. To address this, MDA-MB-231 cells were transfected with tRF-Cys-GCA-029 mimic or its NC. We then performed polysome fractionation experiments to separate the actively translating mRNAs that were associated with polysomes (heavy fraction) from the inactively translating mRNAs that were bounded with monosomes (light fraction) in either tRF-Cys-GCA-029-overexpression or NC cells. We found that tRF-Cys-GCA-029 overexpression could inhibit the translation profile, as indicated by a decrease in polysome content in tRF-Cys-GCA-029-overexpressing cells (Fig. 7C). qRT-PCR was used to quantify the abundance of PRKCG mRNA in the heavy and light fractions. Compared with NC, overexpression of tRF-Cys-GCA-029 increased the level of PRKCG mRNA in monosomes but decreased the abundance of PRKCG mRNA in the polysomes, suggesting that tRF-Cys-GCA-029 suppressed PRKCG translation (Fig. 7D). To quantify how much PRKCG mRNA shifted between the heavier and lighter fractions, we calculated the relative PRKCG mRNA abundance between the lighter and heavier polysomal fractions. The results showed that tRF-Cys-GCA-029 overexpression decreased the polysome/monosome ratio of PRKCG expression, indicating a downregulation of PRKCG translation (Fig. 7E). These data suggest that tRF-Cys-GCA-029 modifies the translation of PRKCG via regulating the abundance of polysomal PRKCG mRNA in BC cells.

To verify whether the endogenous PRKCG protein in BC cells was regulated by tRF-Cys-GCA-029, MCF-7 and MDA-MB-231 cells were transfected with tRF-Cys-GCA-029-mimic and its inhibitor, respectively. And PRKCG protein levels were examined by western blot. The results showed that PRKCG protein levels were down-regulated by tRF-Cys-GCA-029, while tRF-Cys-GCA-029 inhibitor increased the protein levels of PRKCG in BC cells (Fig. 7F-I). Taken together, our results indicate that tRF-Cys-GCA-029 directly regulates PRKCG translation in BC cells.

## Discussion

While accumulating evidence have indicated that preexisting diabetes is associated with poor prognosis in patients with BC [7–10, 30, 31], the underlying mechanisms by which co-existing diabetes drives BC progression remain obscure. In this study, we found that tRF-Cys-GCA-029 was significantly downregulated in BC-DM. We confirmed that hyperglycemia inhibited the expression of tRF-Cys-GCA-029 in BC cells. We revealed that inactivated tRF-Cys-GCA-029 promoted BC cells proliferation and migration in a glucose level-dependent fashion. We further showed that administration of tRF-Cys-GCA-029 suppressed BC tumor growth in diabetic mice. Notably, we discovered that downregulation of tRF-Cys-GCA-029 promoted aerobic glycolysis of BC cells, suggesting that tRF may contribute to BC-DM progression through reprogramming glycolysis metabolism. To the best of our knowledge, this is the first report showing the role of tRF in BC-DM malignancy. Our findings suggest that tRF-Cys-GCA-029 is a BC-DM-associated tRF and targeting tRF-Cys-GCA-029 may be a potential therapeutic approach for BC-DM.

It has been shown that tRFs are selectively produced in response to various stress conditions such as hypoxia, UV radiation, heat shock, and oxidative stress [32]. For example, tRNAGlu, tRNAAsp, tRNAGly, and tRNATyr were upregulated under hypoxia and these tRFs could suppress the progression of breast cancer cells [20]. Oxidative stress exposures increased the production of tRNA (Leu)(TAA) [33]. Ultraviolet (UV) radiation up-regulated the expression of 4 tRFs (tRF-Val-AAC-012, tRF-Pro-AGG-012, tRF-Val-CAC-018, tRF-Val-AAC-031) but down-regulated the generation of other tRFs (tRF-Arg-CCT-002, tRF-Trp-TCA-001, tiRNA-Ser-GCT-001, tRF-Gly-CCC-019, tRF-Ala-TGC-001, tRF-Ala-TGC-002) [34]. Nevertheless, relatively less is known about the impact of glucose levels on tRF generation. Recently, it was observed that tRF-3001a and tRF-5014a significantly upregulated under hyperglycemia conditions [19, 35]. Whereas the expression levels of tRF‑1:30‑Gln‑CTG‑4 and tRF-1020 were decreased in response to high glucose exposure [36, 37]. In the current study, we demonstrated that tRF-Cys-GCA-029 was markedly downregulated under diabetic and hyperglycemia conditions. Moreover, the impact of tRF-Cys-GCA-029 on breast cancer cell proliferation and migration were significantly stronger under hyperglycemic conditions than that under lower glucose conditions. These findings suggest that glucose level may play a critical role in regulating the biogenesis of tRFs. Further investigation is needed to unravel the exact mechanism how glucose regulates the biogenesis of tRFs in BC cells.

Increasing studies have highlighted the critical roles of tRFs in cancer pathogenesis through regulating a variety of biological processes such as cell proliferation and apoptosis, gene expression, ribosome genesis, translation efficiency, and epigenetics [13, 14, 38]. Nevertheless, the effects of tRFs on metabolic reprogramming, which is considered a hallmark of cancer [39, 40], are still poorly understood. A recent study suggested that tRFLys − CTT − 010 was an oncogenic tRF in human triple-negative breast cancer (TNBC) and positively regulated the expression of the starch and sucrose metabolic pathway gene G6PC, which promoted lactate production from breast cancer cells [41]. These data suggested that tRF may be involved in the regulation of glycolysis metabolism. However, the molecular mechanism by which tRF modifies cancer cell malignancy via aerobic glycolysis remains to be investigated. Here, we provided evidence that inactivation of tRF-Cys-GCA-029 led to increased glycolysis metabolism, as indicated by increased production of pyruvate and lactate, as well as higher level of ECAR in BC cells. In contrast, upregulation of tRF-Cys-GCA-029 suppressed aerobic glycolysis in BC cells. In addition, we clarified that tRF-Cys-GCA-029 contributed to BC cell glycolysis metabolism by regulating the expression and translation of PRKCG. Taken together, these analyses suggest that tRF-regulated glycolysis reprograming may play an important role in the progression of BC-DM.

PRKCG (Protein Kinase C Gamma) gene is localized at chromosome 19q13.4.2 and encodes the PKC (Protein Kinase C) enzyme, i.e., PKCγ in humans [42]. PKCγ belongs to the large family of PKC serine/threonine-specific protein kinases which are involved in various cellular and signal transduction pathways [43]. Genetic variants of PRKCG or dysregulation of PKCγ have been implicated in human cancers [44]. However, research on PRKCG gene in the context of breast cancer is scarce. It is unclear whether PRKCG may have pathological significance in BC cells. Herein, we demonstrated that downregulation of tRF-Cys-GCA-029 enhanced PRKCG expression and translation. Furthermore, upregulation of PRKCG promoted cell proliferation and glycolytic metabolism of breast cancer cells. Importantly, rescue experiments and functional assays confirmed that upregulation of PRKCG partially abolished the inhibitory effects of tRF-Cys-GCA-029 on proliferation and glycolysis of BC cells. Thus, our data established the pathological significance of PRKCG in mediating tRF-Cys-GCA-029-induced cell proliferation and Warburg effect in BC cells. Targeting tRF-Cys-GCA-029-PRKCG axis could be an attractive approach for the treatment of BC-DM.

## Conclusions

In summary, a global profiling of tRFs in BC-DM tumor tissues allowed us to identify tRF-Cys-GCA-029 as a key downregulated tRF in BC-DM. Furthermore, we reveal that hyperglycemia-induced downregulation of tRF-Cys-GCA-029 promotes BC cell proliferation and glycolysis through interacting with PRKCG. Importantly, we show that administration of tRF-Cys-GCA-029 suppresses BC tumor growth in diabetic-associated BC. Our findings provide a theoretical basis for the development of potential targeted therapy against BC-DM.

### Electronic supplementary material

Below is the link to the electronic supplementary material.

Supplementary Material 1: The expression profiles of tRFs in tumor tissues of BC-DM and BC-no-DM. (**A**) The volcano plot shows the differentially expressed tRFs between tumor tissues of BC-DM and BC-no-DM. (**B**) Principal component analysis (PCA) of tRFs expressions in BC-DM and BC-no-DM tumor tissues. (**C**) tRFs that are commonly expressed in both BC-DM and BC-no-DM groups and specifically expressed in each group. (**D**) Percentage of tRF subtypes in BC-DM tumor tissues. (**E**) Percentage of tRF subtypes in BC-no-DM tumor tissues. (**F**) Length distribution of tRF subtypes in BC-DM and in BC-no-DM groups. (**G**) The tRF subtype distributions in in different tRNAs in BC-DM and BC-no-DM groups. (**H**) Length distribution of tRF subtypes in BC-DM and in BC-no-DM groups

Supplementary Material 2: Characteristics of tRF-Cys-CGA-29. (**A**) The tRF-Cys-CGA-29 is a type of tRF-1 derived from pre_tRNA-Cys-GCA-2-4, the gene encoding pre_tRNA-Cys-GCA-2-4 is located on chromosome chr17. (**B**) The secondary structure of tRNA-Cys-GCA-2-4. (**C**) The sequence of tRF-Cys-CGA-29.

Supplementary Material 3: Transfection efficiency of synthesized oligonucleotides in BC cells. (**A**) Transfection efficiency of tRF-Cys-CGA-29 mimic and tRF-Cys-CGA-29-inhibitor in MDA-MB-231 cells. (**B**) Transfection efficiency of tRF-Cys-CGA-29 mimic and tRF-Cys-CGA-29-inhibitor in MCF-7 cells. (**C**) Transfection efficiency of tRF-Cys-CGA-29 mimic and tRF-Cys-CGA-29-inhibitor in BT-549 cells

Supplementary Material 4: tRF-Cys-CGA-29 marginally affects apoptosis phenotypes of BC cells. (**A**) Over-expression of tRF-Cys-CGA-29 slightly increases apoptosis rate of MDA-MB-231 cells. (**B**) Inhibition of tRF-Cys-CGA-29 suppresses apoptosis rate of MDA-MB-231 cells

Supplementary Material 5: tRF-Cys-CGA-29-regulated genes are abundant in metabolism-related biological processes

Supplementary Material 6: Transfection efficiency of PRKCG vector in BC cells. (**A**) Transfection efficiency of PRKCG vector on PRKCG gene expression in MDA-MB-231 cells. (**B**) Transfection efficiency of PRKCG vector on PRKCG mRNA expression in MCF-7 cells. (**C**) Transfection efficiency of PRKCG vector on PRKCG protein expression in MDA-MB-231 cells. (**D**) Transfection efficiency of PRKCG vector on PRKCG protein expression in MCF-7 cells. ***P < 0.01, ****P < 0.001

Supplementary Material 7

Supplementary Material 8

Supplementary Material 9

Supplementary Material 10

Supplementary Material 11

Supplementary Material 12

Supplementary Material 13

## Data Availability

No datasets were generated or analysed during the current study.

## Abbreviations

tRNA: Transfer RNA
tRF: tRNA-derived fragment
BC-DM: Breast cancer with diabetes
TPM: Total aligned tRNA reads
ECAR: Extracellular acidification rate
PCA: Principal component analysis
HRP: Horseradish peroxidase
PVDF: Polyvinylidene fluoride
SDS-PAGE: Sodium Dodecyl Sulphate-Polyacrylamide Gel Electrophoresis
CCK-8: Cell Counting Kit-8
PRKCG: Protein Kinase C Gamma
qRT-PCR: Quantitative real time PCR
DEG: Differentially expressed gene
FDR: False discovery rate
GO: Gene Ontology
KEGG: Kyoto Encyclopedia of Genes and Genomes
GSEA: Gene Set Enrichment Analysis
CDS: Coding sequence
