# Supplementary figures and images for: Downregulation of tRF-Cys-GCA-029 by hyperglycemia promotes tumorigenesis and glycolysis of diabetic breast cancer through upregulating PRKCG translation
Source: Breast Cancer Res. 2024 Jul 22;26:117. doi: 10.1186/s13058-024-01870-1 (PMC11265092; doi:10.1186/s13058-024-01870-1)

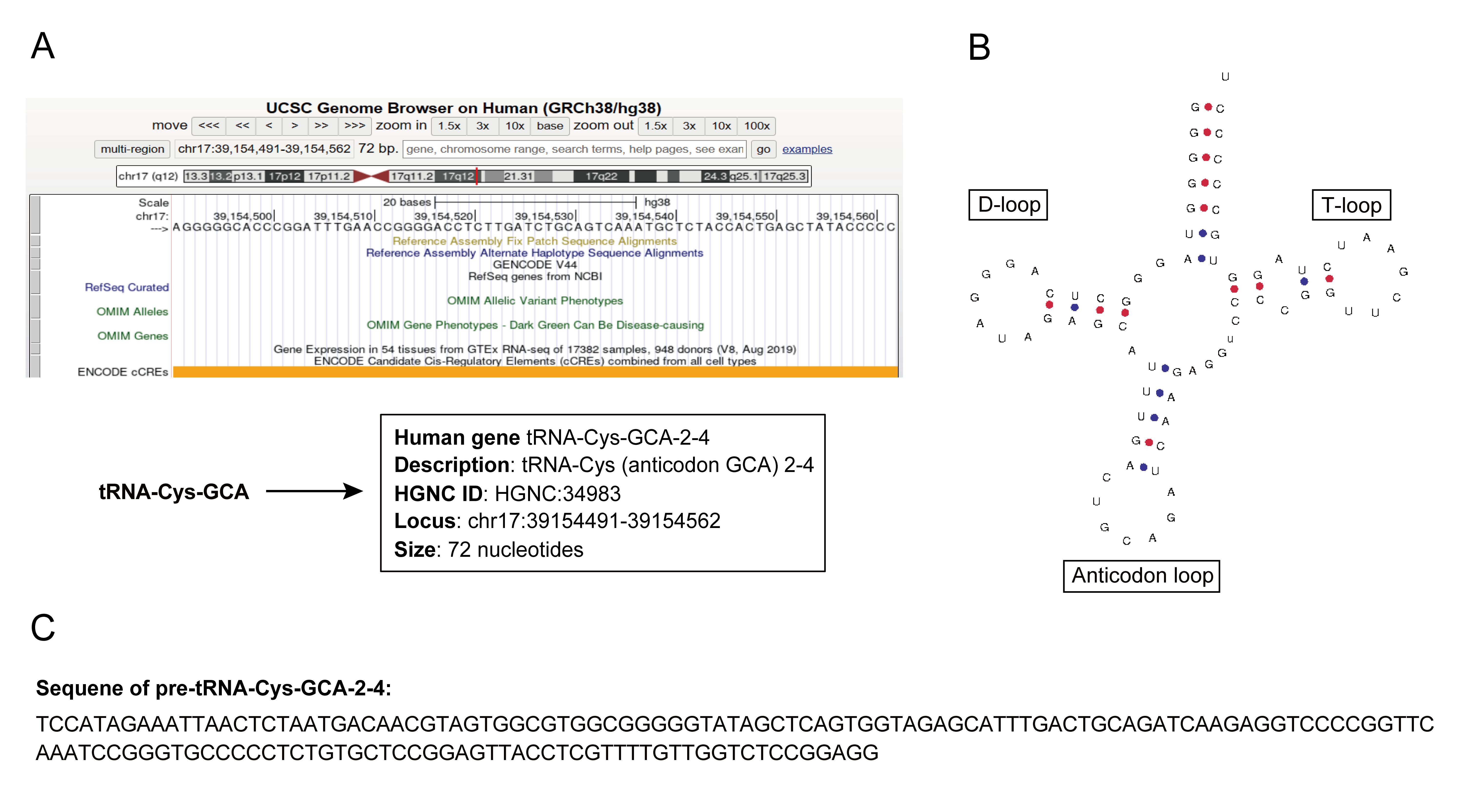

Supplement: Supplementary file 2 — Supplementary Material 2: Characteristics of tRF-Cys-CGA-29. (A) The tRF-Cys-CGA-29 is a type of tRF-1 derived from pre_tRNA-Cys-GCA-2-4, the gene encoding pre_tRNA-Cys-GCA-2-4 is located on chromosome chr17. (B) The secondary structure of tRNA-Cys-GCA-2-4. (C) The sequence of tRF-Cys-CGA-29. [file 13058_2024_1870_MOESM2_ESM.tif]

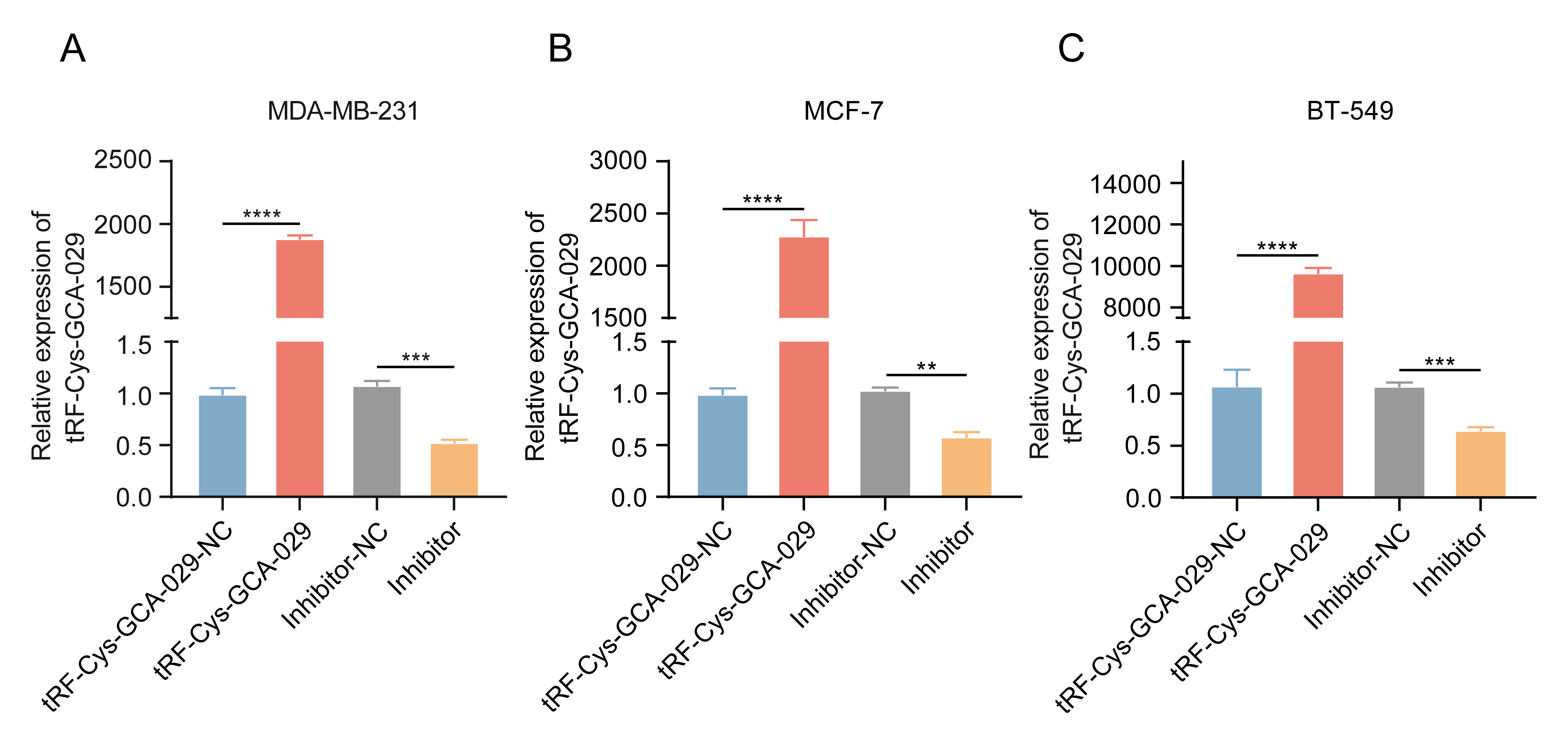

Supplement: Supplementary file 3 — Supplementary Material 3: Transfection efficiency of synthesized oligonucleotides in BC cells. (A) Transfection efficiency of tRF-Cys-CGA-29 mimic and tRF-Cys-CGA-29-inhibitor in MDA-MB-231 cells. (B) Transfection efficiency of tRF-Cys-CGA-29 mimic and tRF-Cys-CGA-29-inhibitor in MCF-7 cells. (C) Transfection efficiency of tRF-Cys-CGA-29 mimic and tRF-Cys-CGA-29-inhibitor in BT-549 cells [file 13058_2024_1870_MOESM3_ESM.tif]

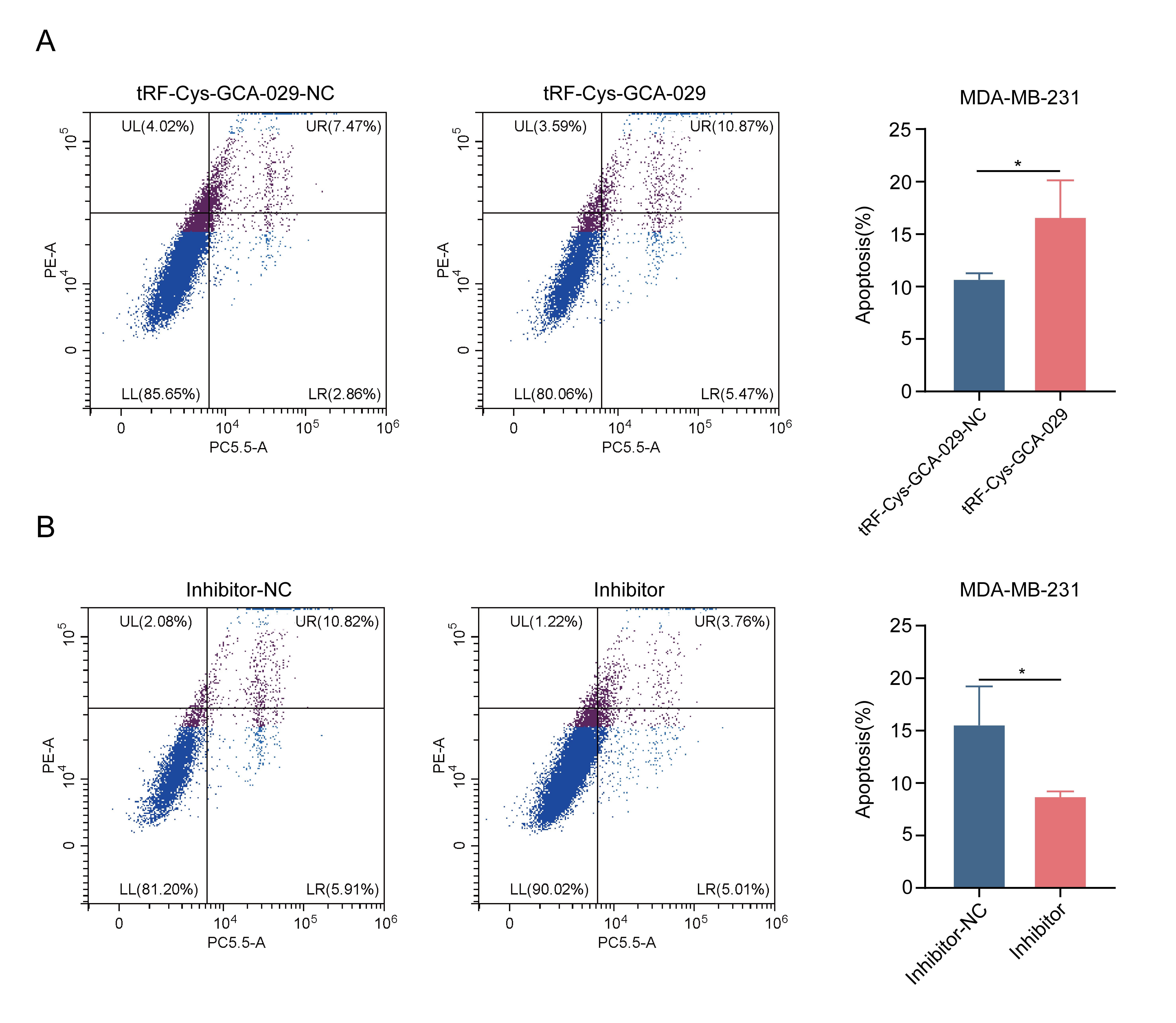

Supplement: Supplementary file 4 — Supplementary Material 4: tRF-Cys-CGA-29 marginally affects apoptosis phenotypes of BC cells. (A) Over-expression of tRF-Cys-CGA-29 slightly increases apoptosis rate of MDA-MB-231 cells. (B) Inhibition of tRF-Cys-CGA-29 suppresses apoptosis rate of MDA-MB-231 cells [file 13058_2024_1870_MOESM4_ESM.tif]

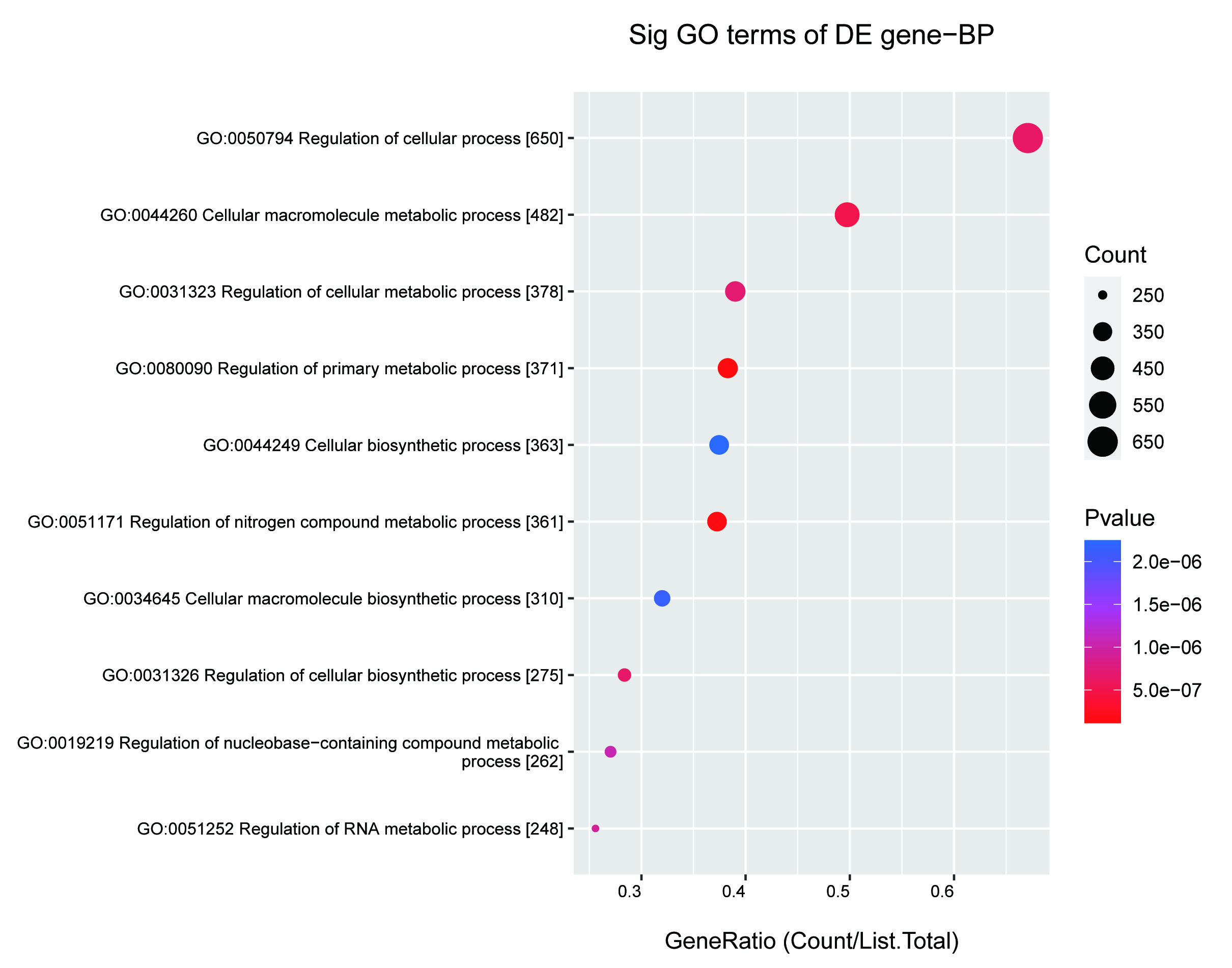

Supplement: Supplementary file 5 — Supplementary Material 5: tRF-Cys-CGA-29-regulated genes are abundant in metabolism-related biological processes [file 13058_2024_1870_MOESM5_ESM.tif]

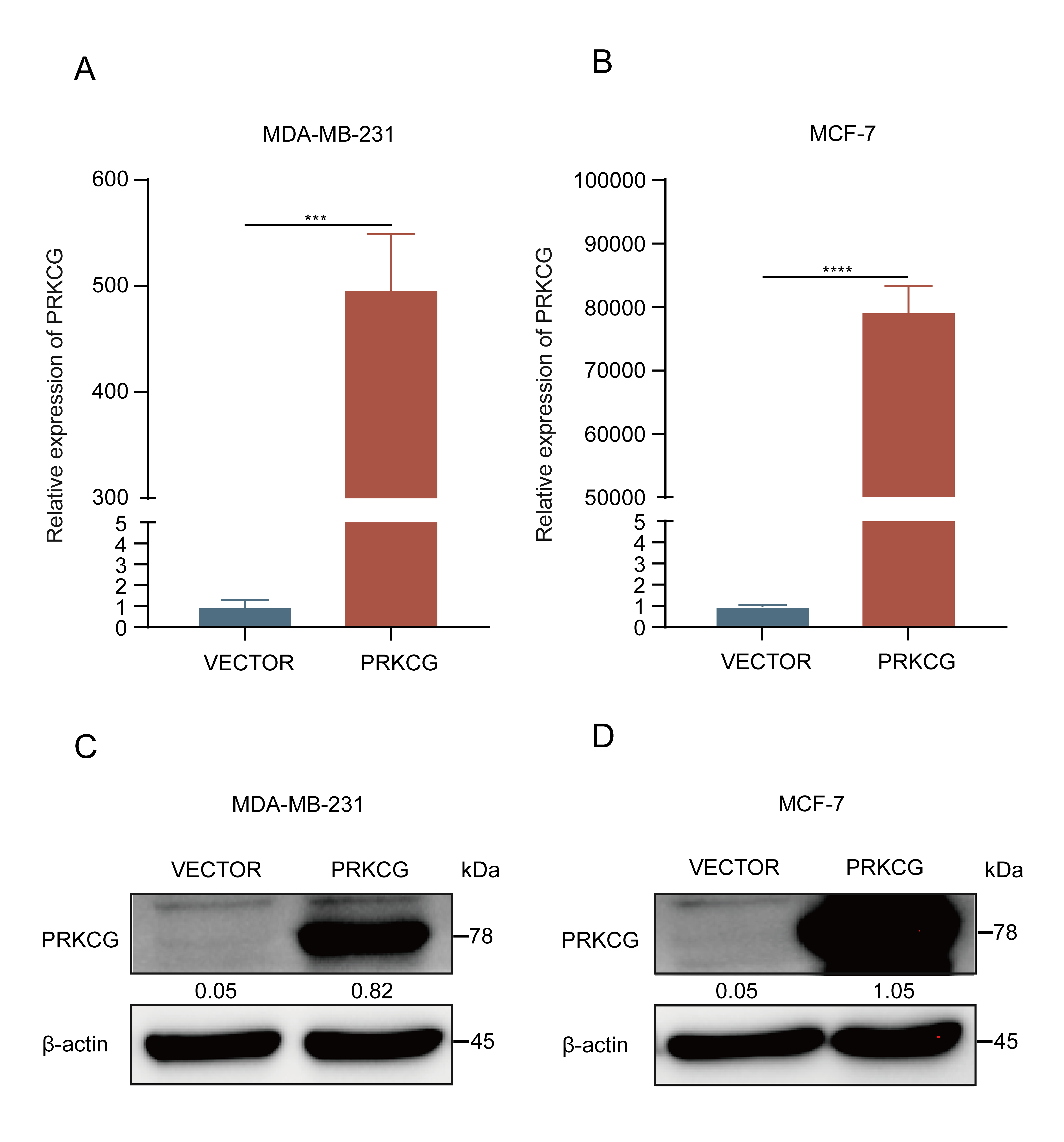

Supplement: Supplementary file 6 — Supplementary Material 6: Transfection efficiency of PRKCG vector in BC cells. (A) Transfection efficiency of PRKCG vector on PRKCG gene expression in MDA-MB-231 cells. (B) Transfection efficiency of PRKCG vector on PRKCG mRNA expression in MCF-7 cells. (C) Transfection efficiency of PRKCG vector on PRKCG protein expression in MDA-MB-231 cells. (D) Transfection efficiency of PRKCG vector on PRKCG protein expression in MCF-7 cells. ***P < 0.01, ****P < 0.001 [file 13058_2024_1870_MOESM6_ESM.tif]

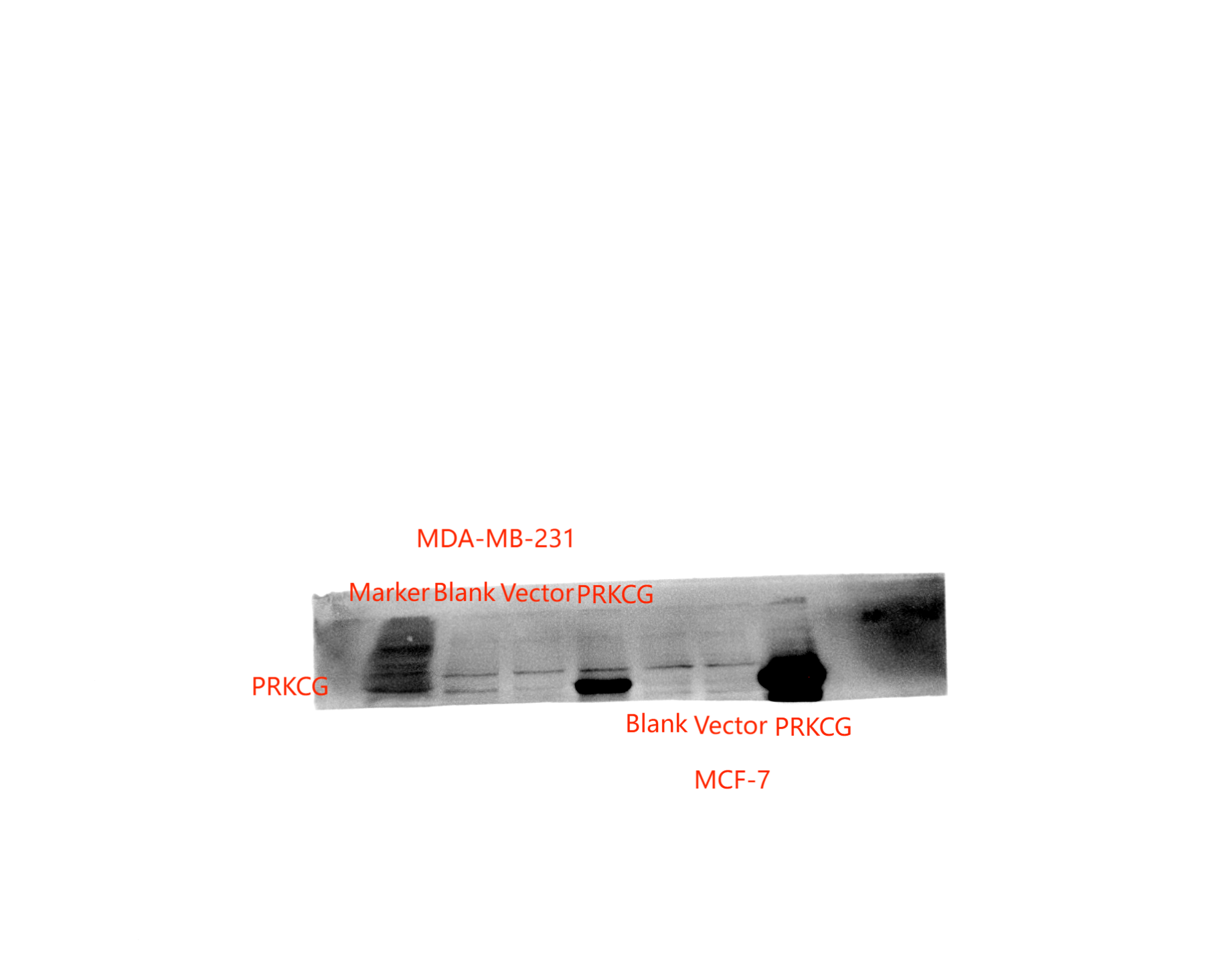

Supplement: Supplementary file 7 — Supplementary Material 7 [file 13058_2024_1870_MOESM7_ESM.png]

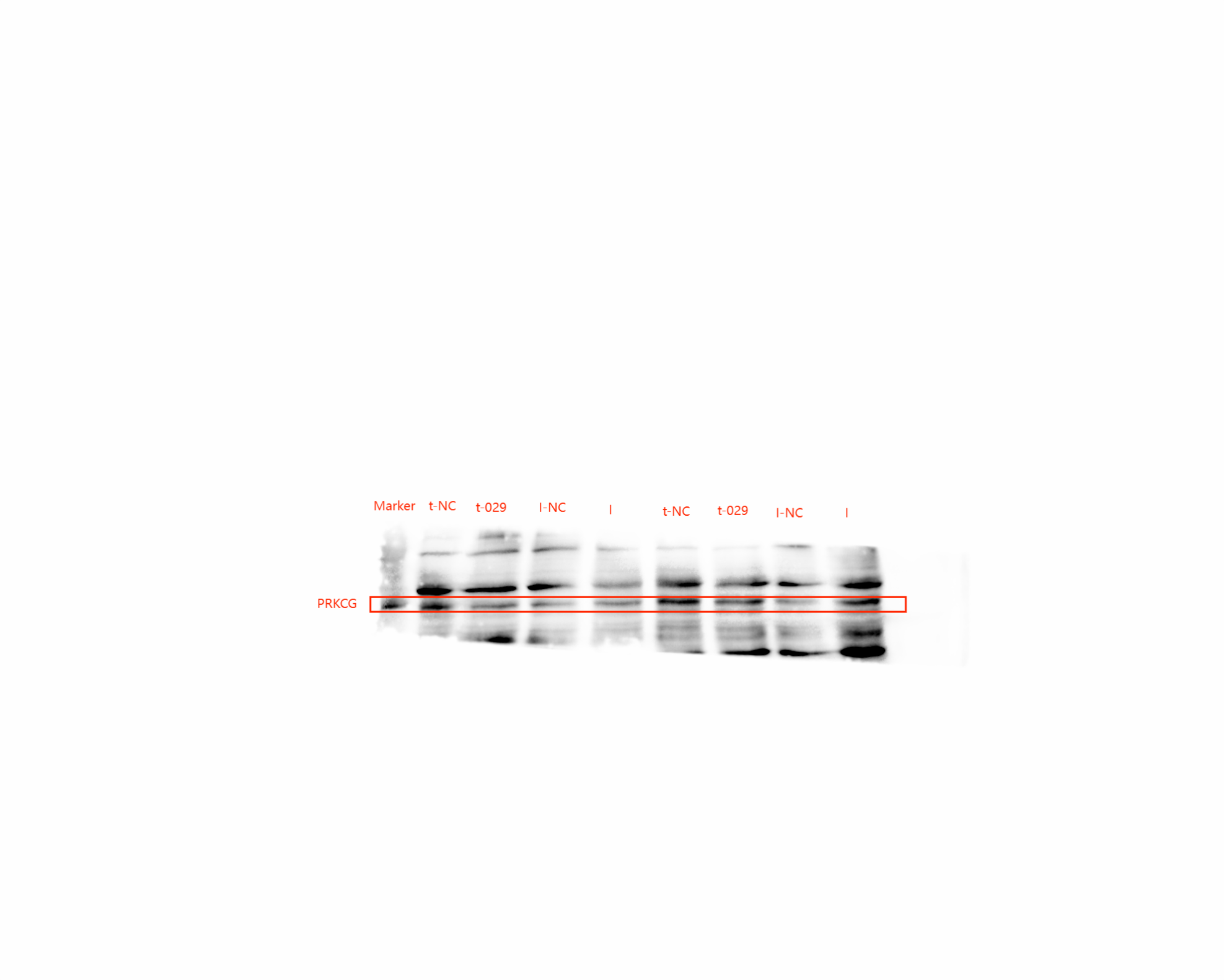

Supplement: Supplementary file 8 — Supplementary Material 8 [file 13058_2024_1870_MOESM8_ESM.png]

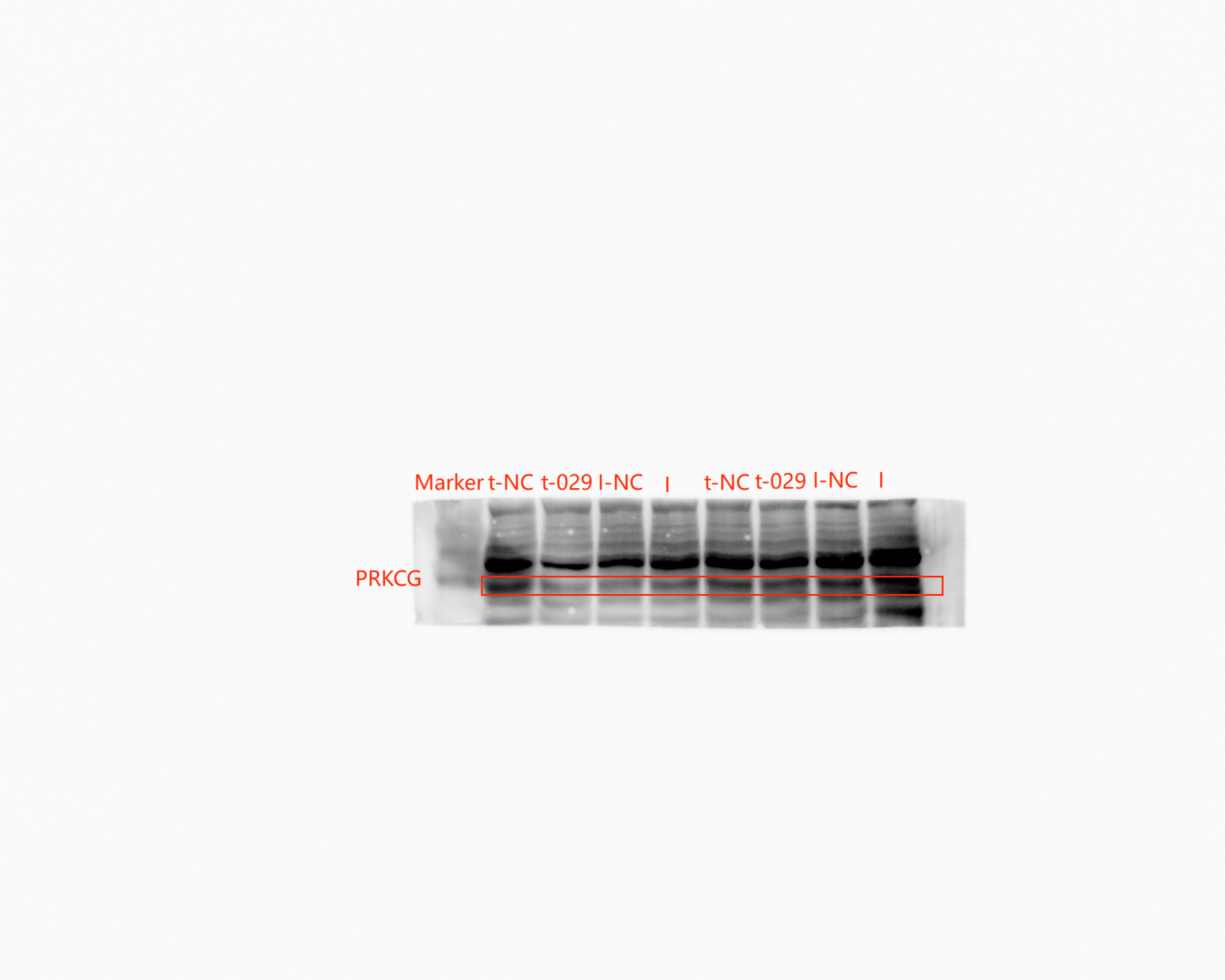

Supplement: Supplementary file 9 — Supplementary Material 9 [file 13058_2024_1870_MOESM9_ESM.png]

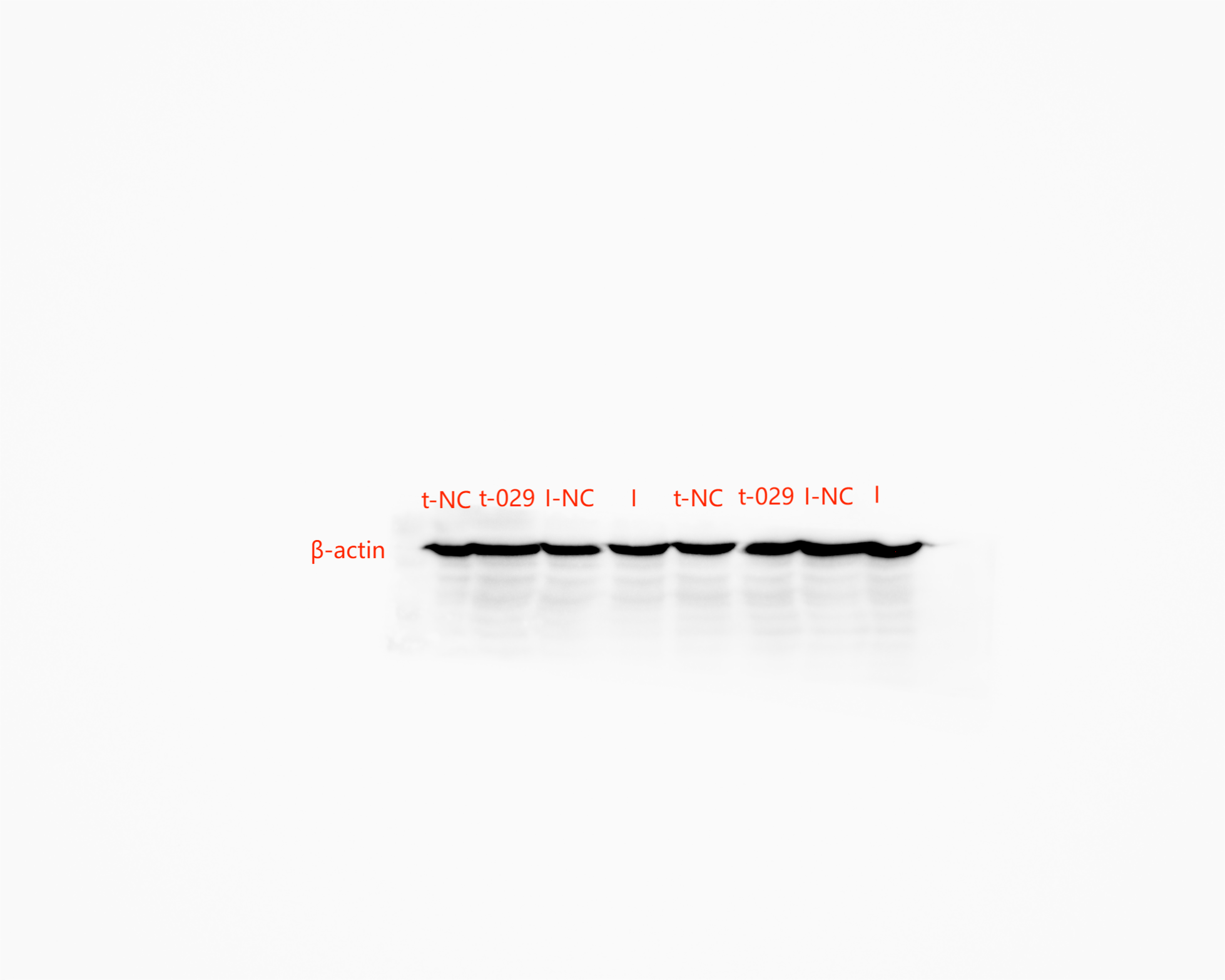

Supplement: Supplementary file 10 — Supplementary Material 10 [file 13058_2024_1870_MOESM10_ESM.png]

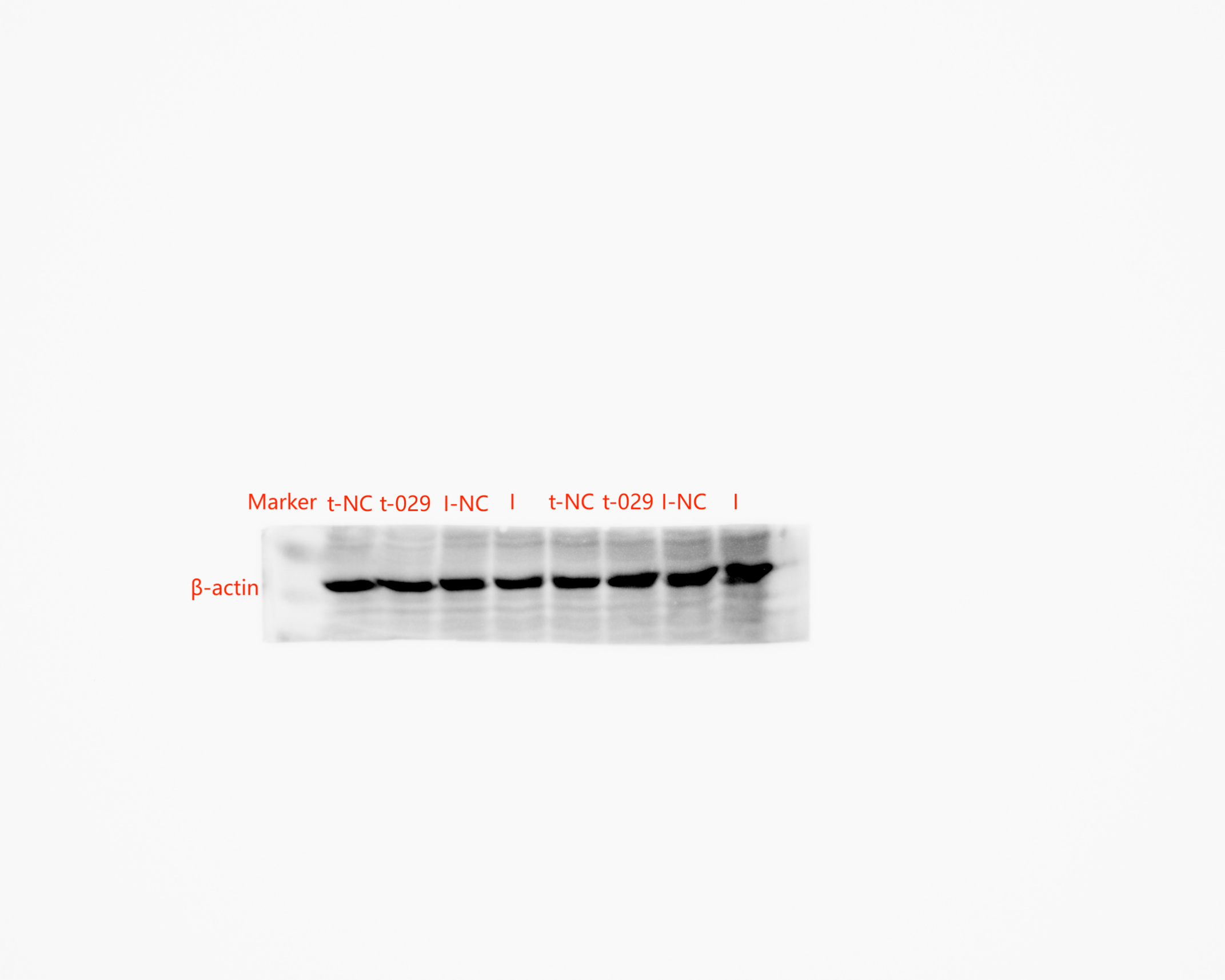

Supplement: Supplementary file 11 — Supplementary Material 11 [file 13058_2024_1870_MOESM11_ESM.png]

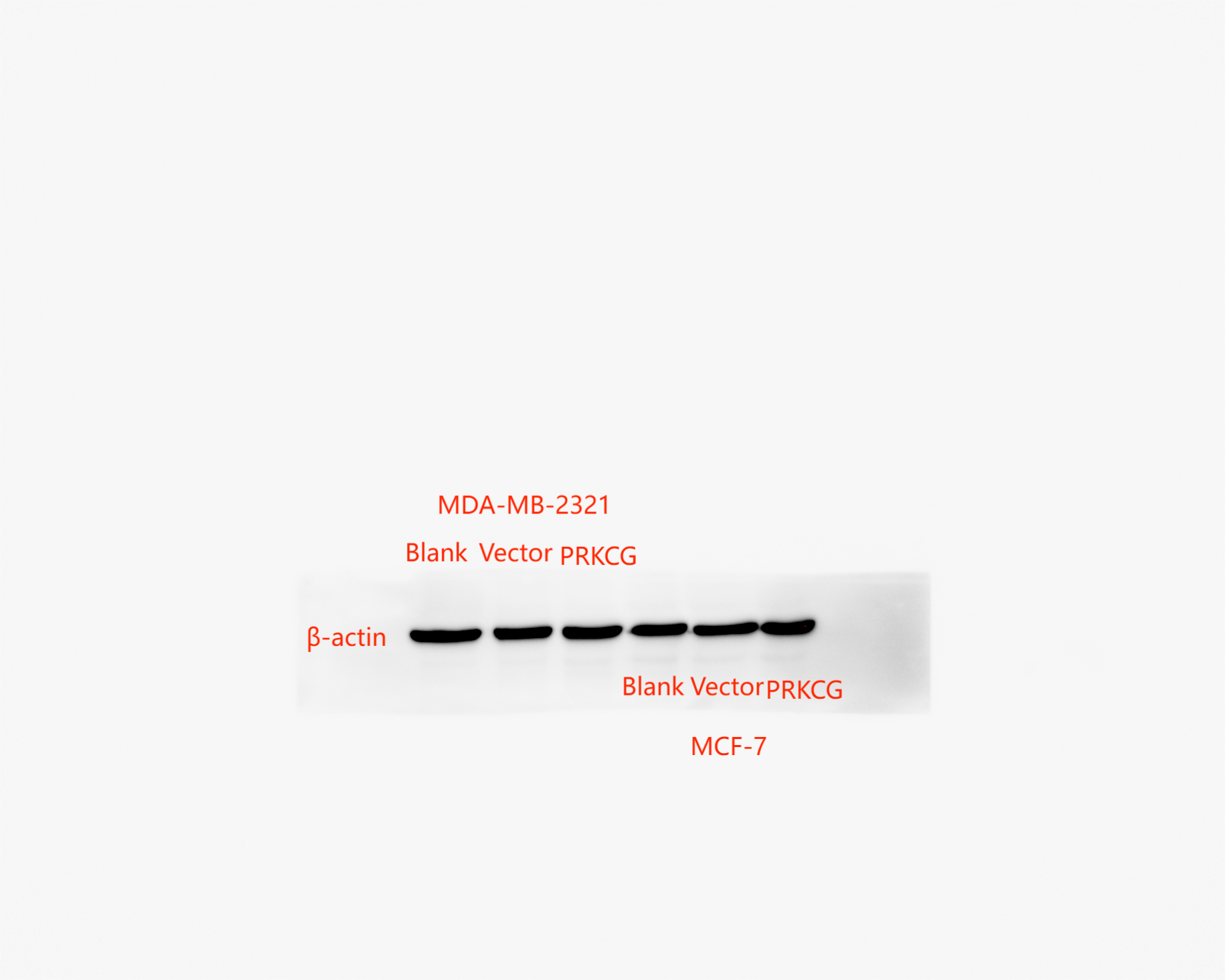

Supplement: Supplementary file 12 — Supplementary Material 12 [file 13058_2024_1870_MOESM12_ESM.png]
