# Supplementary material for: Downregulation of tRF-Cys-GCA-029 by hyperglycemia promotes tumorigenesis and glycolysis of diabetic breast cancer through upregulating PRKCG translation
Source: Breast Cancer Res. 2024 Jul 22;26:117. doi: 10.1186/s13058-024-01870-1 (PMC11265092; doi:10.1186/s13058-024-01870-1)
Supplement: Supplementary file 13 — Supplementary Material 13 [file 13058_2024_1870_MOESM13_ESM.docx]

| Table S1. Characteristics of patients with BC-DM or BC-no-DM selected for tumor tissue tRF sequencing (Harbin samples) | | | | | | | | | | | |  |
| --- | --- | --- | --- | --- | --- | --- | --- | --- | --- | --- | --- | --- |
| Sample ID | Sex | Marital status | Age | Fasting blood-glucose (mmol/L) | BMI, kg/m2 | Menopausal status | Stage | ER status (%) | PR status (%) | HER-2 status | Ki-67 status (%) | Use of anti-diabetic drug |
| BC01 | Female | Married | 46 | 5.5 | 24.1 | No | I | 90 | 90 | 0 | 25 | No |
| BC02 | Female | Married | 52 | 5.5 | 28.5 | Yes | I | 90 | 40 |  | 10 | No |
| BC03 | Female | Married | 60 | 6.3 | 25.3 | Yes | I | 80 | 70 | 0 | 25 | No |
| BC04 | Female | Married | 47 | 6.3 | 23.4 | No | II | 90 | 0 | 0 | 15 | No |
| BC05 | Female | Married | 45 | 6.5 | 27.5 | No | I | 80 | 50 | 0 | 15 | No |
| BC06 | Female | Married | 72 | 4.7 | 27.1 | Yes | II | 10 | 0 | 0 | 80 | No |
| BC-DM1 | Female | Married | 51 | 7.8 | 29 | Yes | I | 90 | 50 | 0 | 10 | Glichite |
| BC-DM2 | Female | Married | 61 | 8.5 | 29.3 | Yes | I | 90 | 70 | 0 | 10 | Insulin |
| BC-DM3 | Female | Married | 62 | 14.4 | 28.6 | Yes | I | 60 | 0 | 0 | 15 | Insulin |
| BC-DM4 | Female | Married | 46 | 8.2 | 24.7 | No | I | 0 | 0 | 0 | 15 | Metformin |
| BC-DM5 | Female | Married | 49 | 12.7 | 24.6 | No | II | 0 | 0 | 1 | 8 | Insulin |
| BCD-M6 | Female | Married | 55 | 16.4 | 27 | Yes | I | 80 | 15 | 1 | 40 | Pioglitazone, Glipizide |

| Table S2. Characteristics of patients with BC-DM or BC-no-DM selected for qRT-PCR analysis (Shenzhen samples) | | | | | | | | | | | |  |
| --- | --- | --- | --- | --- | --- | --- | --- | --- | --- | --- | --- | --- |
| Sample ID | Sex | Marital status | Age | Fasting blood-glucose (mmol/L) | BMI, kg/m2 | Menopausal status | Stage | ER status (%) | PR status (%) | HER-2 status | Ki-67 status (%) | Use of anti-diabetic drug |
| BC01 | F | M | 77 | 5.8 | 23 | Y | IIIA | 10 | - | 3+ | 50 | N |
| BC02 | F | M | 49 | 5.38 | 20.4 | N | IIB | 90 | 80 | 1+ | 8 | N |
| BC03 | F | M | 46 | 5.34 | 19.9 | Y | IIA | - | - | 0 | 80 | N |
| BC04 | F | M | 58 | 5.31 | 26.7 | Y | IA | 90 | 80 | 0 | 15 | N |
| BC05 | F | M | 42 | 4.43 | 20.57 | N | IIA | 90 | 80 | 0 | 40 | N |
| BC06 | F | M | 44 | 3.45 | 17.63 | N | IIA | 85 | 80 | 3+ | 40 | N |
| BC07 | F | M | 55 | 5.05 | 23.63 | Y | IIA | 80 | 30 | 3+ | 55 | N |
| BC08 | F | M | 46 | 4.35 | 21.09 | N | IIA | 1 | 2 | 3+ | 80 | N |
| BC-DM1 | F | M | 54 | 14.32 | 22.9 | Y | IIA | - | - | 3+ | 50 | N |
| BC-DM2 | F | M | 38 | 8.45 | 27.5 | N | IIA | 80 | 90 | 0 | 80 | N |
| BC-DM3 | F | M | 38 | 11.45 | 21.6 | N | IIA | - | - | 3+ | 20-30 | N |
| BC-DM4 | F | M | 67 | 8.47 | 24 | Y | IIA | - | - | - | 60 | Y |
| BC-DM5 | F | M | 65 | 8.24 | 20.2 | Y | IIIC | / | / | / | / | N |
| BC-DM6 | F | M | 36 | 7.89 | 29.7 | N | IIB | 90 | - | 1+ | 1 | N |
| BC-DM7 | F | M | 71 | 7.16 | 23.7 | Y | IIB | 2 | - | 1+ | 10-15 | N |
| BC-DM8 | F | M | 64 | 5.63 | 23.61 | Y | IIIB | 90 | 90 | 2+ | 10 | Y |

**Table S2 The sequence information for relevant RNAs, Related to STAR Methods**

| Name |  |  |
| --- | --- | --- |
| tRF-Cys-GCA-029 mimic | CTGTGCTCCGGAGTTACCTCGTTTT | |
| Mimic NC | UCUCGGUCCAUUCUAGGUUCGUUGC | |
| tRF-Cys-GCA-029 inhibitor | AAAACGAGGUAACUCCGGAGCACAG | |
| Inhibitor NC | GCAACGAACCUAGAAUGGACCGAGA | |
| Primers for PRKCG | Forward: 5’AGCCACAAGTTCACCGCTC3’  Reverse: 5’ GGACACTCGAAGGTCACAAAT3’ | |
| PRKCG mimic  pmirGLO-PRKCG-Site1-WT | CTTCCCCTAGTCCCACCGACCCCAAGCGCTGCTTCTTCGGGGCGAGTCCAGGACGCCTGCACATCTCCGACTTCAGCTTCCTCATGGTTCTAGGAAAAGG | |
| pmirGLO-PRKCG-Site1-Mut | CTTCCCCTAGTCCCACCGACCCCAAGCGCTGCTTCTGATTTTATCTGAACTTCATAAGTACACGCTCCGACTTCAGCTTCCTCATGGTTCTAGGAAAAGG | |
| pmirGLO-PRKCG-Site2-WT | GACCAAGCACCCAGGGAAGCGCCTGGGCTCAGGGCCTGATGGGGAACCTACCATCCGTGCACATGGCTTTTTCCGCTGGATTGACTGGGAGCGGCTGGAA | |
| pmirGLO-PRKCG-Site2-Mut | GACCAAGCACCCAGGGAAGCGCCTGGGCTCAGGGCCGTCGTTTTCCAAGCAACGAATGTACACGGGCTTTTTCCGCTGGATTGACTGGGAGCGGCTGGAA | |

**Table S3.** Antibodies, reagents and chemicals

| Reagent or resource | Source | Identifier |
| --- | --- | --- |
| Antibodies |  |  |
| anti-PRKCG | Proteintech | 14364-1-AP |
| anti-β-actin | Abcam | ab101562 |
| horseradish peroxidase (HRP) conjugated β-actin secondary antibodies | ZSGB-BIOZS | ZB-5305 |
| Chemicals, reagents |  |  |
| Lipofectamine 3000 | Life Technologies | Cat#L3000015 |
| Lipofectamine 2000 | Invitrogen | 11668-027 |
| STZ | Yeasen | Cat#60256ES |
| Tripsin | Gibco | Cat#25200-072 |
| 0.25% tripsin | Gibco | Cat#11965118 |
| DMEM | Gibco | Cat#11965092 |
| RPMI 1640 | Gibco | Cat#11875093 |
| FBS | BIOAGRIO | Cat#S1001-500 |
| Trizol | Life Technologies | Cat#15596026 |
| CCK-8 | Dojiindo | Cat#CK04 |
| Transwell chamber | Falcon | Cat#3422 |
| FITC Annexin V Apoptosis Detection Kit | BD Biosciences | Cat#556547 |
| Dual-Luciferase Reporter Assay System | Promega | E1910 |
| Crystal violet | Beyotime | Cat#C0121 |
| RNA6000 Labchip Kit | Agilent | Cat#5067-1511 |
| L-lactate assay kit | Jiancheng Bioengineering | Cat#A019-2-1 |
| Pyruvate assay kit | Jiancheng Bioengineering | Cat#A081-1-1 |
| Glycolytic stress test kit | Seahorse | Cat#103020–100 |
| High fat diet | Boaigang | 1135DM-5 |
| NextSeq 500/550 V2 kit | Illumina | #FC-404-2005 |
| Triton-X 100 | Beyotime | Cat#P0096 |
| Tris | MACKLIN | Cat#T818967 |
| KCl | MACKLIN | Cat#P816355 |
| MgCl2 | MACKLIN | Cat#:M813763 |
| DTT | Meilunbio | Cat#MB3047 |
| CHX | J&K Scientific | Cat#917321 |
| Heparin | Solarbio | Cat#H8060 |

**Table S4.** Softwares and bioinformatics tools

| Software and Database |  |  |
| --- | --- | --- |
| Perl | http://www.perl.org | v5.16.3 |
| Python | https://www.python.org | 2.7.5 |
| R | https://www.r-project.org | 3.5.1 |
| FastQC | http://www.bioinformatics.babraham.ac.uk | v0.11.7 |
| Cutadapt | http://cutadapt.readthedocs.io/en/stable/index.html | 1.17 |
| Bowtie | http://bowtie-bio-sourceforge.net/index.shtml | 1.2.2 |
| miRDeep2 | https://www.mdc-berlin.de | 2.0.0.8 |
| GtRNAdb | http://gtrnadb.ucsc.edu/ | N/A |
| tRFdb | http://genome.bioch.virginia.edu/trfdb/ | N/A |
| MINIbase | http://cm.jefferson.edu/MINTbase/ | N/A |
| miRBase | http://www.mirbase.org/index.shtml. | N/A |
| GO | http://www.geneontology.org | N/A |
| KEGG | http://www.genome.jp/kegg/ | N/A |
| GSEA | http://software.broadinstitute.org/gsea/index.jsp | 2.2.4 |
| Hisat2 | http://ccb.jhu.edu/software/hisat2/index.shtml | 2.1.0 |
| StringTie | http://ccb.jhu.edu/software/stringtie/shtml | 1.3.3 |
| TargetScan | http://www.targetscan.org | N/A |
| miRanda | http://www.microrna.org | N/A |
